# Genetic polymorphisms of non-coding RNAs associated with increased head and neck cancer susceptibility: a systematic review and meta-analysis

**DOI:** 10.18632/oncotarget.20096

**Published:** 2017-08-09

**Authors:** Weiyi Pan, Chenzhou Wu, Zhifei Su, Zexi Duan, Longjiang Li, Fanglin Mi, Chunjie Li

**Affiliations:** ^1^ State Key Laboratory of Oral Diseases, West China Hospital of Stomatology, Sichuan University, Chengdu, China; ^2^ Department of Head and Neck Oncology, West China Hospital of Stomatology, Sichuan University, Chengdu, China; ^3^ Department of Stomatology, Affiliated Hospital of North Sichuan Medical College, Sichuan, China

**Keywords:** genetic polymorphisms, non-coding RNAs, head and neck cancer, cancer susceptibility, systematic review

## Abstract

Genetic polymorphisms, including single nucleotide polymorphisms (SNP) and nucleotide repeat expansions, can occur in regions that transcribe non-coding RNAs (ncRNA), such as, but not limited to, micro RNA and long non-coding RNA. An association between genetic polymorphisms of ncRNA and increasing head and neck cancer (HNC) risk has been identified by several studies. Therefore, the aim of this systematic review is to consolidate existing findings to clarify this association. Four electronic databases, such as MEDLINE, EMBASE, Chinese BioMedical Literature Database, and China National Knowledge Infrastructure, were utilised. Inclusion of studies and data extraction were accomplished in duplicate. A total of 42 eligible studies were included, involving 28,527 cases and 37,151 controls. Meta-analysis, sensitivity analysis and publication bias detection were performed. Among the eligible studies, 102 SNPs were investigated, and 21 of them were considered eligible for meta-analysis. Our analysis revealed that HOTAIR rs920778, uc003opf.1 rs11752942, and miR-196a2 rs11614913 were related to HNC susceptibility, while let-7 rs10877887, miR-124-1rs531564, and miR-608 rs4919510 were considered as protective factors. In conclusion, our results showed the extreme importance of an up-to-date comprehensive meta-analysis encompassing the most recent findings to obtain a relevant and reliable framework to understand the relationship between ncRNA SNPs and HNC susceptibility.

## INTRODUCTION

Head and neck cancer (HNC) is a broad term encompassing epithelial malignancies located in the oral cavity, pharynx, larynx, cervical oesophagus, paranasal sinuses, nasal cavity, salivary glands and thyroid gland [1–3]. The anatomical sites affected by HNC are of great importance in several functions such as speech, smell, swallowing and chewing. Thus, HNC and tumour removal can greatly impact quality of life [1, 3]. According to the GLOBOCAN 2008 study, HNC is the sixth most common type of cancer worldwide, accounting for an estimated number of 650,000 new cancer cases and 350,000 cancer deaths every year [3, 4]. It is worth noting that only a third of HNC patients present at the early stage of disease, but after appropriate treatments, the cure rate can reach up to 90% in patients with stage I disease and about 70% in patients with stage II disease [3]. Thus, HNC patients can benefit from early diagnosis and treatment. Identification of the risk factors contributing to HNC susceptibility may be helpful for early management. Oral tobacco use (smoking and betel quid chewing), alcohol consumption, viral infections (Epstein-Barr virus and human papilloma virus), dental hygiene, and dietary factors are proven risk factors for HNC [5–7]. In addition to the above environmental factors, a role of genetic factors in HNC cancer development has also been confirmed [8]. Recently, genetic polymorphisms in genes encoding non-coding RNAs (ncRNAs) have been linked with HNC risk.

Pervasive transcription of human genomes has been demonstrated with the development of whole genome technologies. Interestingly, only 2% of transcripts encode proteins, while most transcripts are ncRNAs [8–11]. ncRNA can be functionally divided into the following three subgroups: 1) ‘housekeeping’ ncRNAs, including ribosomal RNA, transfer RNA, small nuclear RNA and small nucleolus RNA, 2) regulatory ncRNAs, including micro RNAs (miRNAs), long non-coding RNAs (lncRNAs), small interfering RNAs, and PIWI-interacting RNAs, and 3) several other poorly characterized types of ncRNAs [11, 12]. The most important cancer-related ncRNAs include the broadly investigated miRNAs, as well as the recently recognized lncRNAs [12].

MiRNAs are small ncRNAs of 21–23 nucleotides, which use partial base-pairing to recognize target messenger RNAs, thus repressing their expression [13]. Hence, miRNAs play an important role in several fields including angiogenesis, epithelial–mesenchymal transition and drug resistance by targeting various oncogenes and tumour suppressors in cancer cells [13, 14]. lncRNAs are defined as transcripts longer than 200 nucleotides with little or no open reading frames [15]. The regulatory mechanisms of lncRNAs are much more complicated than that of miRNAs, and include chromatin reprogramming, post-transcriptional regulation, protein localization, RNA interference [16, 17]. Although the exact biological role of most lncRNAs remain unclear, there is increasing evidence to suggest that lncRNAs are associated with carcinogenesis and act as an important component of tumour biology [17, 18].

Despite physical differences, human populations are 99% genetically identical, which means that human diversity, including cancer susceptibility, results from the remaining 1% of genetic polymorphisms [19–21]. Small- and large-scale genetic polymorphisms, including single nucleotide polymorphisms (SNPs), nucleotide expansions, copy-number alterations, and chromosomal translocations, can occur in non-coding regions of the genome [22]. Most of these genetic polymorphisms are due to SNPs caused by single nucleotide mutations that occur on average approximately once in 100 to 300 base pairs in the human genome [20]. Since Abelson et al. in 2005 firstly demonstrated the association between SNPs in miRNA-related genes and Tourette’s syndrome [23], SNPs in genes encoding ncRNAs have been widely investigated, especially in the field of cancer susceptibility [24–27]. To date, accumulating evidence has revealed a link between ncRNA polymorphisms and HNC susceptibility. To date, there is some evidence showing a link between ncRNA polymorphisms and HNC susceptibility. However, the results of epidemiological studies have been inconsistent. Therefore, the aim of this systematic review was to consolidate existing findings to clarify this association.

## RESULTS

### Study identification

The process of study identification was showed in Supplementary Figure 1.

After the removal of duplicates, 193 records were identified through electronic database searching and manual searching. One hundred and thirty-one records were excluded after the screening of the abstract, while the full texts of the remaining 62 records were analysed to confirm their eligibility. Among these 62, 20 were excluded, such as three studies in which primary head and neck cancer was actually not diagnosed, seven studies that did not investigate ncRNA polymorphisms, six studies that did not analyse the association between genetic polymorphism and cancer risk, and four studies that did not report sufficient data for statistical analysis. Finally, 42 studies [28–69] were considered eligible for this meta-analysis and included.

### Study characteristics

Among the 42 included studies, six studies [28, 43, 46, 58, 62, 64] contained multiple cohorts, thus were considered as independent studies. For this reason, a total of 50 comparisons were included in this systematic review, which investigated 102 different polymorphisms in a large number of cases and controls amounting 28,527 and 37,151, respectively. Regarding ncRNAs genetic polymorphisms, only studies that investigated lncRNAs and miRNAs SNPs were found. The ethnic background of the participants included Asian [30, 32, 33, 35–38, 42, 44–51, 53–59, 61–69], Black [43], Caucasian [28, 31, 34, 39–41, 52, 60], and mixed [29, 43]. Several cancer types were investigated, such as oesophageal cancer [30, 32, 42–44, 46, 47, 49, 53, 55, 57, 58, 60, 63, 65, 68], head and neck squamous cell carcinoma (HNSCC) [29, 31, 35, 40], nasopharyngeal carcinoma (NPC) [36, 38, 48, 50, 64], oral squamous cell carcinoma (OSCC) [33, 37, 41, 51, 52, 54, 59, 62], and thyroid carcinoma [28, 34, 39, 45, 56, 61, 66, 67, 69]. It is important to mention that four studies [29, 41, 46, 60] did not completely report the distribution of three genotypes, although, these data were extracted and analysed as well. All included studies were case control studies. The remaining characteristics of the included studies were listed in Supplementary Table 1.

After the screening of the full text and data extraction, 21 out of 102 SNPs were considered eligible for meta-analysis. Among them, two SNPs were located into lncRNAs, and the rest were located into miRNAs. The control group of nine cohorts from six studies [33, 43, 44, 59, 65, 66] were not in agreement with HWE (*P* > 0.05). Except all the three cohorts of miR-219-1 rs213210 and two of the four cohorts of miR-26a-1: rs7372209, the rest cohorts were of different SNPs (listed in detail in Supplementary Table 1). The detailed information of these SNPs was listed in Table 1, while the SNPs that were not eligible for meta-analysis were listed in Supplementary Table 2.

**Table 1:** Characteristics of SNPs that eligible for meta-analysis

ncRNA	SNP	Ancestral Allele	SNP Alleles	Ethnic	Cancer type	Number of Studies *	References
**lncRNAs**							
**HOTAIR**	rs920778	C	C/T	Asian	ESCC	3	[58]
**uc003opf.1**	rs11752942	A	A/G	Asian	ESCC	2	[46]
**miRNAs**							
**let-7**	rs10877887	T	T/C	Asian	OSCC,PTC	2	[59, 66]
**let-7**	rs13293512	T	T/C	Asian	OSCC,PTC	2	[59, 66]
**miR-26a-1**	rs7372209	C	T/C	Asian, Black, Mixed	ESCC	4	[42–44]
**miR-27a**	rs895819	T	T/C	Asian	ESCC	2	[44, 57]
**miR-34b/c**	rs4938723	T	T/C	Asian	ESCC, NPC, PTC	4	[36, 47, 57, 61]
**miR-124-1**	rs531564	C	G/C	Asian	ESCC	2	[47, 57]
**miR-146a**	rs2910164	G	C/G	Asian, Caucasian	ESCC, HNSCC, LSCC, NPC, OSCC, TC	20	[28, 30, 31, 33, 34, 38-41, 42,44, 45, 48, 51, 52, 53, 67, 69]
**miR-149**	rs2292832	C	T/C	Asian, Caucasian	ESCC, OSCC, PTC	5	[31, 33, 35, 41, 56]
**miR-196a2**	rs11614913	C	C/T	Asian, Caucasian, Mixed	ESCC, HNSCC, NPC, OSCC, PTC	13	[29, 31–33, 37, 41, 42, 44, 50,53–55, 67]
**miR-218-2**	rs11134527	G	G/A	Asian	ESCC	2	[49, 57]
**miR-219-1**	rs213210	T	T/C	Asian, Black, Mixed	ESCC	3	[43, 65]
**miR-423**	rs6505162	C	A/C	Asian, Black, Mixed	ESCC	4	[42, 43, 47]
**miR-449b**	rs10061133	A	A/G	Asian	ESCC, PTC	2	[67, 68]
**miR-499a**	rs3746444	T	T/C	Asian, Caucasian	ESCC, HNSCC, NPC, OSCC, PTC	9	[31, 33, 41, 42, 44, 62,64, 67]
**miR-608**	rs4919510	G	C/G	Asian	ESCC, NPC, PTC	4	[64, 67, 68]
**miR-627**	rs2620381	A	A/C	Asian	ESCC, PTC	2	[67, 68]
**miR-646**	rs6513497	T	T/G	Asian	ESCC, PTC	2	[67, 68]
**miR-3152**	rs13299349	G	G/A	Asian	NPC, PTC	2	[64, 67]
**miR-4293**	rs12220909	G	G/C	Asian	ESCC, NPC, PTC	3	[64, 67, 68]

*: the cohorts would be considered as different studies if more than one cohort were reported separately in one single study.

ncRNA: noncoding RNAs; SNPs: single nucleotide polymorphisms; lncRNAs: long noncoding RNAs; miRNAs: micro RNAs; ESCC: esophageal squamous cell carcinoma; HNSCC: head and neck squamous cell carcinoma; NPC: nasopharyngeal carcinoma; OSCC: oral squamous cell carcinoma; PTC: papillary thyroid carcinoma; LSCC: laryngeal squamous cell carcinoma; TC: thyroid carcinoma.

The result of methodological quality assessment by Newcastle–Ottawa quality assessment scale (NOS) of included studies was listed in Supplementary Table 1.Among the 50 cohorts from 42 included studies, two [34, 54] were considered as high risk of bias, 19 [28, 33, 35, 37–39, 43, 48, 49, 52, 56, 60, 61, 63, 67, 69] were considered as medium risk of bias, and the rest 29 were considered as low risk of bias.

### Meta-analysis results

**Figure 1:**
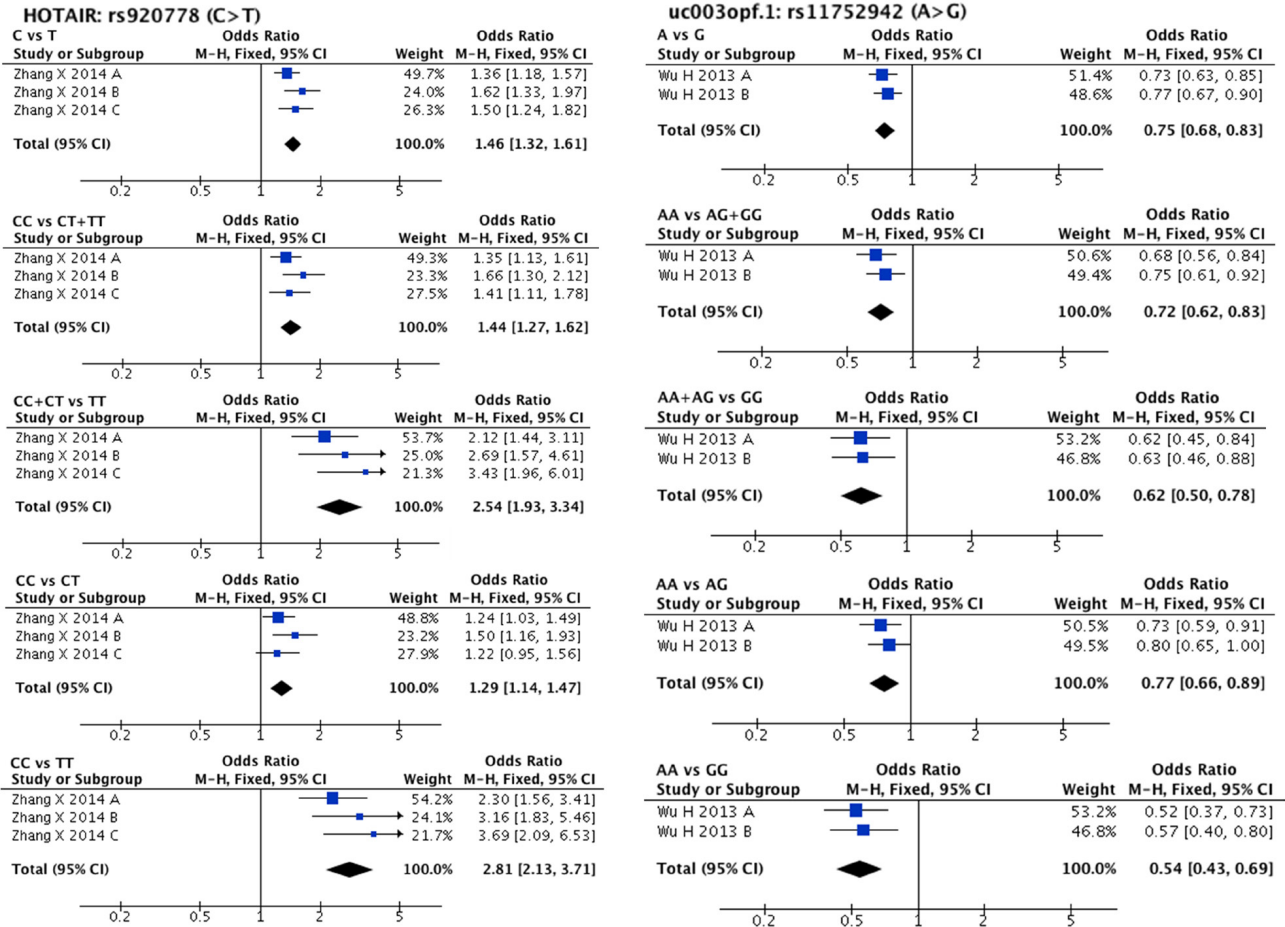
Forest plots of effect estimates for two lncRNAs HOTAIR rs920778 and uc003opf.1 rs11752942 Five forest plots of each lncRNA are corresponded to five genetic models (allele contrast, dominant model, recessive model, and two co-dominant models).

Meta-analysis results are listed in Supplementary Table 4. lncRNA SNPs, HOTAIR rs920778 (C/T) and uc003opf.1 rs11752942 (A/G) were considered eligible for meta-analysis. For HOTAIR rs920778, three comparisons were found in a single study including 2098 cases and 2150 controls, and for uc003opf.1 rs11752942, two comparisons were found also in a single study including 1493 cases and 1553 controls, respectively [46, 58]. A significant association was found in all genetic models between oesophageal squamous cell carcinoma (abbreviated to ESCC to differentiate it from oral squamous cell carcinoma) susceptibility and both HOTAIR rs920778 (C allele vs. T allele: OR = 1.46, 95% CI = 1.32–1.61, P-H = 0.37; CC vs. CT + TT: OR = 1.44, 95% CI = 1.27–1.62, P-H = 0.40; CC + CT vs. TT:OR = 2.54, 95% CI = 1.93–3.34, P-H = 0.37; CC vs. CT:OR = 1.29, 95% CI = 1.14–1.47, P-H = 0.43; CC vs. TT:OR = 2.81, 95% CI = 2.13–3.71, P-H = 0.36) and uc003opf.1 rs11752942 (A allele vs. G allele: OR = 0.75, 95% CI = 0.68–0.83, P-H = 0.57; AA vs. AG + GG: OR = 0.72, 95% CI = 0.62–0.83, P-H = 0.50; AA + AG vs. GG: OR = 0.62, 95% CI = 0.50–0.78, P-H = 0.91; AA vs. AG: OR = 0.77, 95% CI = 0.66–0.89, P-H = 0.53; AA vs. GG: OR = 0.54, 95% CI = 0.43–0.69, P-H = 0.74). The forest plot of the comparisons above is shown in Figure 1. We did not perform subgroup analysis since no clinical and statistical heterogeneity were found.

A total of 19 SNPs found into miRNAs gene loci were included into meta-analysis. The overall pooled result showed a significant association between miRNA SNP and increased HNC risk only in 1 SNP, such as miR-196a2 rs11614913 (C/T), in co-dominant model (CC vs. CT: OR = 1.12, 95% CI = 1.03–1.22, P-H = 0.07), which was supported by 13 comparisons (7107 cases and 7233 controls). Furthermore, we found that several SNPs acted as protective factors according to the overall pooled result in certain genetic models, such as let-7 rs10877887 (T/C; TT vs. TC + CC: OR = 0.80, 95% CI = 0.68–0.95, P-H = 0.81;TT vs. TC: OR = 0.77, 95% CI = 0.64–0.92, P-H = 0.54),miR-124-1 rs531564(C/G; CC + CG vs. GG: OR = 0.46, 95% CI = 0.28–0.75, P-H = 0.31; CC vs. GG: OR = 0.45, 95% CI = 0.27, 0.75, P-H = 0.34), and miR-608 rs4919510(G/C; GG + GC vs. CC: OR = 0.82, 95% CI = 0.68–0.98, P-H = 0.07), which were supported by two comparisons (1002 cases and 1293 controls), two comparisons (1738 cases and 1961 controls), and four comparisons (3191 cases and 3899 controls), respectively.

**Figure 2:**
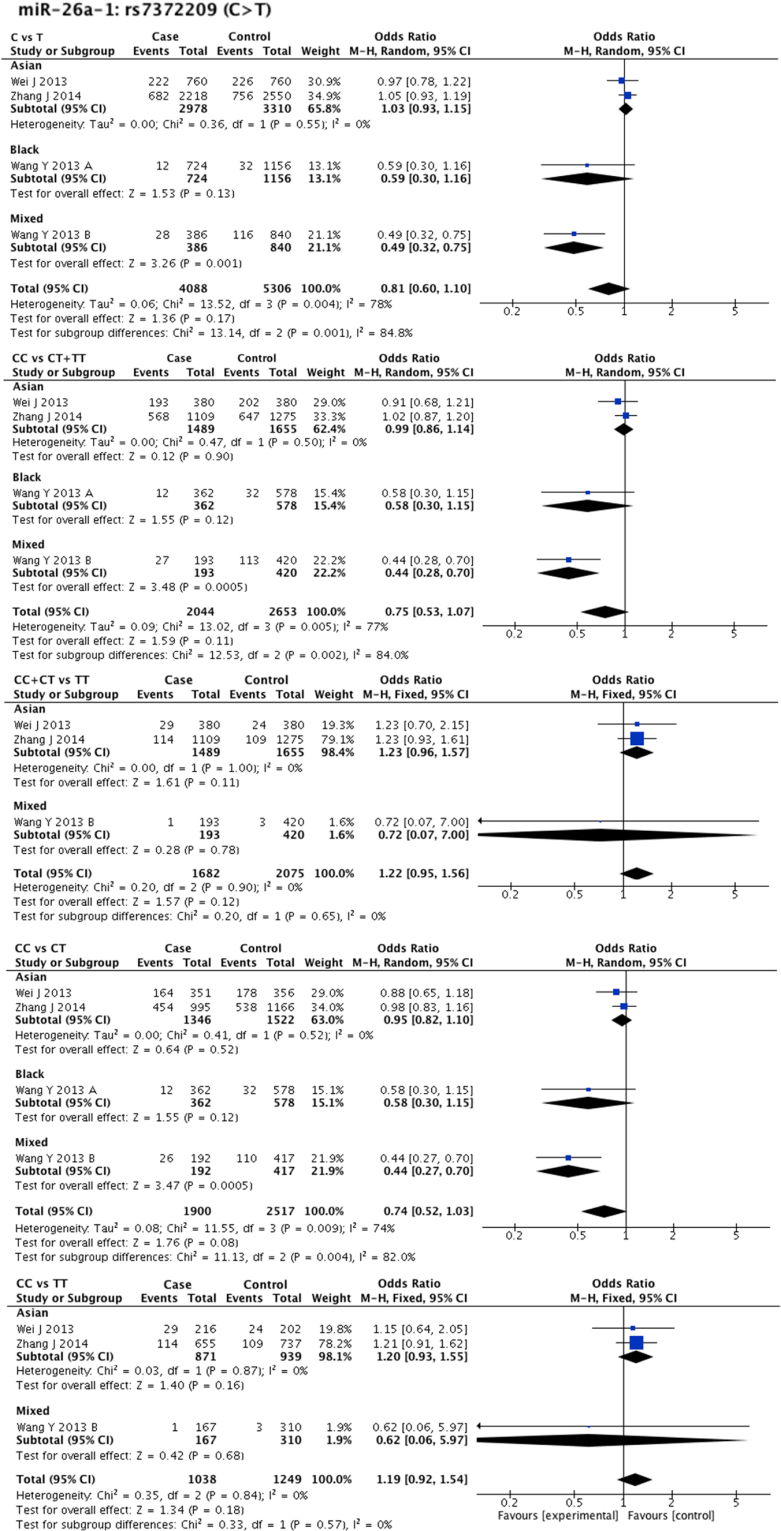
Forest plots of effect estimates for miR-26a-1 Five forest plots of each lncRNA are corresponded to five genetic models. Since all the participants of miR-26a-1 are ESCC, subgroup analysis was only performed based on ethnicity.

**Figure 3:**
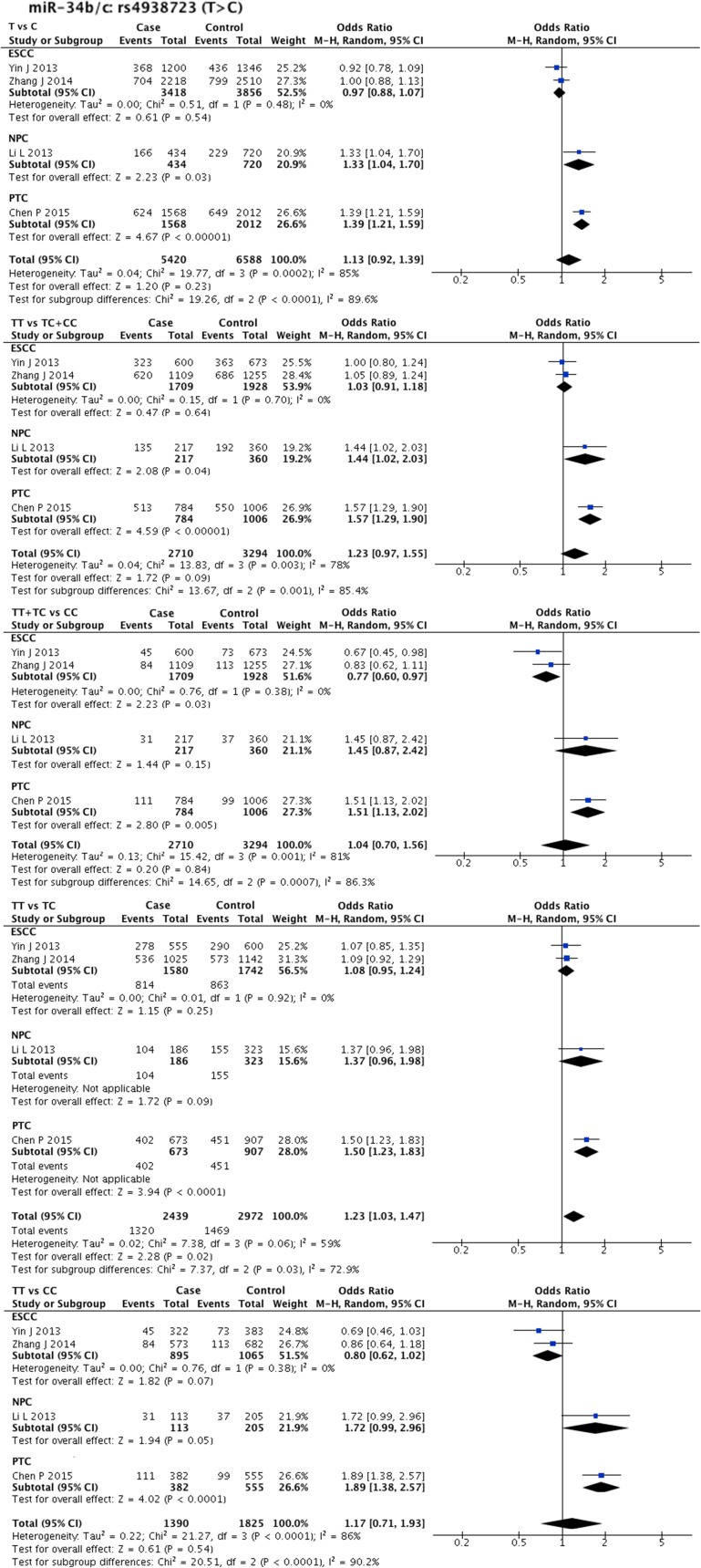
Forest plots of effect estimates for miR-34bc Five forest plots of each lncRNA are corresponded to five genetic models. Since all the participants of miR-34bc are Asian, subgroup analysis was only performed based on the type of cancer. ESCC: esophageal squamous cell carcinoma; NPC: nasopharyngeal carcinoma; PTC: papillary thyroid carcinoma.

**Figure 4:**
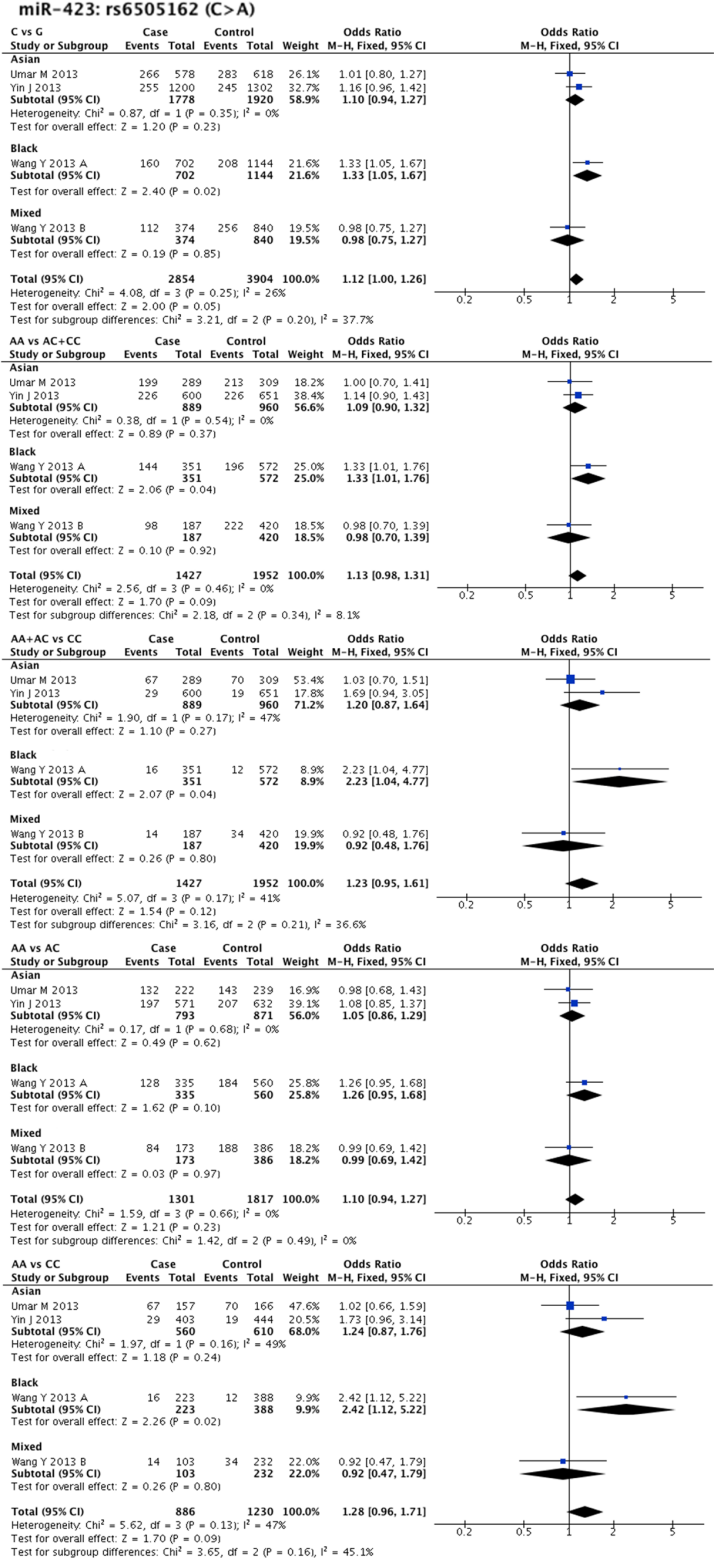
Forest plots of effect estimates for miR-423 Five forest plots of each lncRNA are corresponded to five genetic models. Since all the participants of miR-423 are ESCC, subgroup analysis was only performed based on ethnicity.

**Figure 5:**
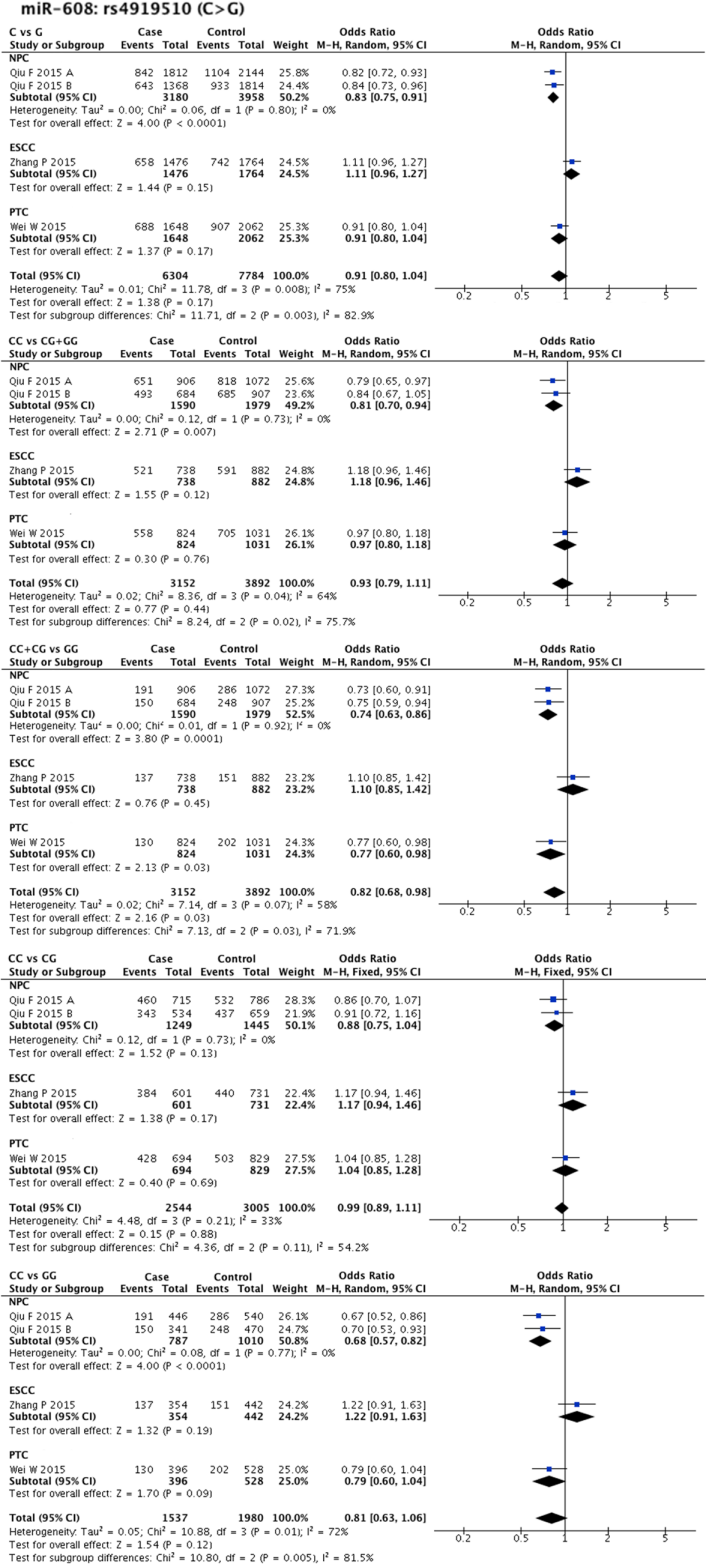
Forest plots of effect estimates for miR-608 Five forest plots of each lncRNA are corresponded to five genetic models. Since all the participants of miR-608 are Asian, subgroup analysis was only performed based on the type of cancer. ESCC: esophageal squamous cell carcinoma; NPC: nasopharyngeal carcinoma; PTC: papillary thyroid carcinoma.

Statistical heterogeneity was found in 11 SNPs. Eight SNPs underwent subgroup analysis to detect the source of heterogeneity, while miR-218-2 rs11134527 (G/A), and miR-27a rs895819 (T/C), miR-449b rs10061133 (A/G) were not subjected to subgroup analysis because the number of included studies was too small(*n* = 2). According to the methods described in methods part, subgroup analysis was performed based on ethnicity and cancer type. The results of subgroup analysis were listed in Supplementary Table 5. The forest plots of subgroup analysis of four SNPs, such as mir-26a-1 rs7372209 (C/T), miR-34b/c rs4938723 (T/C), miR-423 rs6505162 (C/A), and miR-608 rs4919510 (G/C), were shown in Figures 2–5.

MiR-146a rs2910164 (G/C) was found to be related to NPC susceptibility (G allele vs. C allele: OR = 1.39, 95% CI = 1.12–1.72, P-H = 0.88; GG + GC vs. CC: OR = 1.77, 95% CI = 1.31–2.40, P-H = 0.92; GG vs. CC: OR = 1.64, 95% CI = 1.02–2.61, P-H = 0.57), while only two comparisons (393 cases and 373 controls) were included in this subgroup. We also found that miR-196a2 rs11614913T allele could significantly increase HNC risk in Asian populations (CC + CT vs. TT: OR = 1.14, 95% CI = 1.01–1.22, P-H = 0.04) supported by 10 comparisons (5189 cases and 5213 controls). SNP miR-196a2 rs11614913 was also found to be related to OSCC susceptibility by pooled result of four comparisons (1561 cases and 1304 controls). Protective factors were also evaluated in specific ethnic backgrounds or cancer types. SNP miR-608 rs4919510 (G/C) showed protective effect on NPC susceptibility (G allele vs. C allele: OR = 0.83, 95% CI = 0.75–0.91, P-H = 0.8; GG vs. GC + CC: OR = 0.81, 95% CI = 0.70–0.94, P-H = 0.73; GG + GC vs. CC: OR = 0.74, 95% CI = 0.63–0.86, P-H = 0.92; GG vs. CC: OR = 0.68, 95% CI = 0.57–0.82, P-H = 0.77), while only two comparisons (1590 cases and 1979 controls) were included in this subgroup. In addition, miR-499a rs3746444 (T/C) was found to be a protective factor in Caucasian populations (CC vs. CT + TT: OR = 0.80, 95% CI = 0.69–0.94, P-H = 0.54), and contributed to ESCC (C allele vs. T allele: OR = 0.80, 95% CI = 0.66–0.98, P-H = 0.75), as supported by the pooled result of two comparisons (669 cases and 688 controls).

Interestingly, the overall pooled result for miR-34b/c rs4938723 (T/C) differed from the subgroup analysis result. The overall pooled result of four comparisons (2739 cases and 3327 controls) indicated that SNP miR-34b/c rs4938723 (T/C) was associated with HNC susceptibility (TT vs. TC: OR = 1.23, 95% CI = 1.03–1.47, P-H = 0.06), while subgroup analysis showed its protective effect against ESCC (TT + TC vs. CC: OR = 0.77, 95% CI = 0.60–0.97, P-H = 0.38), as supported by pooled result of two comparisons (1738 cases and 1961 controls).

In addition, omitting the cohorts whose control group was not in agreement with HWE did not change the result of meta-analysis (data not shown). Besides, omitting the cohorts that were considered as high risk of bias did not changed the results of meta-analyses (data not shown). The influence of cohort that was considered as medium risk of bias on the pooled result would be discussed in conclusion below.

### Sensitivity analysis and publication bias

No significant change was observed when omitting individual studies in turn, suggesting that the results were reliable. Publication bias of the two SNPs, such as miR-146a rs2910164 and miR-196a2 rs11614913 was detected by funnel plots (Supplementary Figure 2). Asymmetry of the funnel plots of some genetic models was observed, while no significant publication bias was found by Harbord test (Supplementary Table 3).

## DISCUSSION

Identifying genetic markers of cancer susceptibility and gene-environment interactions could contribute to the reduction of cancer mortality through early diagnosis and personalized therapy [20]. Genome-wide association studies (GWAS) have greatly extended our knowledge of SNPs associated with disease phenotypes, and demonstrated that 88% of associated SNPs are located in non-coding regions (either intronic or intergenic) [70, 71]. Since the role of genetic polymorphisms in HNC aetiology has been confirmed, the relationship between SNPs in these non-coding regions and HNC susceptibility has drawn increasing attention from many researchers. However, stronger evidence is needed to validate and further support this relationship. Thus, the purpose of our meta-analysis was to develop a more comprehensive understanding of the changes involved in HNC.

According to a recent study by the ENCODE Project Consortium, approximately 9640 lncRNA gene loci are present in the human genome, while the total number continues to grow [70–73]. The current knowledge related to the interaction between lncRNAs and cancer mainly includes changes in cancer-related lncRNA expression in cancer cells, suggesting that IncRNAs contribute to cancer susceptibility [22]. However, the precise mechanism by which lncRNA polymorphisms are responsible for the oncogenesis remains unknown, since how lncRNA polymorphisms affect the interaction with other biomolecules, such as DNA, protein and other RNA, it is not yet fully understood [22].

A large number of studies have proved that HOTAIR can act as an oncogene [74–76]. HOTAIR rs920778T allele is significantly correlated with increased HOTAIR RNA expression [58] which might result in malignant transformation of normal cells [74–76]. Regarding uc003opf.1 rs11752942, a change from A allele to G allele could significantly decrease lncRNA expression and directly suppress tumour development [46]. In accordance with its biological functions, our meta-analysis results demonstrated that HOTAIR rs920778 (C/T) and uc003opf.1 rs11752942 (A/G) could significantly increase and decrease, respectively, ESCC risk in Chinese populations. However, it is noteworthy that 3 comparisons investigating HOTAIR rs920778, as well as 2 comparisons investigating uc003opf.1 rs11752942, were provided by one single study [46, 58]. Thus, more case-control studies are needed to confirm the relationship between these two SNPs and HNC susceptibility.

Biogenesis and maturation of miRNAs is well depicted by several studies over the past decades [77–83]. Briefly, miRNA gene loci are transcribed by RNA polymerase II in order to synthetize primary miRNAs (pri-miRNAs) transcripts. Pri-miRNAs are then enzymatically cleaved by DROSHA complex to release small hairpins and form stem-loop precursor miRNAs (pre-miRNAs). After moving from the nucleus to the cytoplasm, pre-miRNAs are subsequently converted by the DICER complex to mature miRNAs. Mature miRNAs usually bind to the 3′ untranslated region (UTR), as well as 5′ UTR and coding regions of target mRNAs to mediate translational repression or RNA degradation. On the basis of the above process, SNPs residing in miRNA gene loci may affect one, two or all three steps, such as transcription of the primary transcript, processing of pri-miRNAs and pre-miRNAs, and miRNA–mRNA interactions. The effects above may result in increasing or decreasing of mature miRNAs levels, enhancement or suppression of target gene identification, and even destruction of target binding sites [20]. As miRNAs are functionally associated with oncogenesis and have the ability to simultaneously affect many genes, SNPs in miRNAs could theoretically lead to phenotypic variations that contribute to cancer susceptibility.

Large numbers of systematic reviews have been performed to investigate the association between miRNAs SNPs and cancer susceptibility, including breast cancer, colorectal cancer, gastric cancer, liver cancer, lung cancer [84–90]. In contrast, only few systematic reviews are available on the relationship between HNC susceptibility and miRNAs SNP [2, 91]. Compared to a recently published systematic review [2], a certain number of new studies were added in the meta-analysis of the present systematic review, including 11 new studies investigating mir-146a rs2910164, eight new studies investigating mir-196a rs11614913, six new studies investigating mir-499a rs3746444, and two new studies investigating mir-149 rs2292832. Besides, we also analysed another 15 SNPs residing in miRNA gene loci, which were not included in the meta-analysis by Niu *et al.* [2]*.* Hence, to the best of our knowledge, the present systematic review represents the most comprehensive meta-analysis since it incorporated the most recent studies when compared to other reviews associated to miRNA SNPs and HNC susceptibility relationship.

The SNP rs2910164 in miR-146a is located in the stem region of the precursor opposite to the mature miR-146 sequence, therefore a change from G allele to C allele could affect the stem structure of the miR-146a precursor, thus decreasing mature miRNA expression and suppressing oncogenesis [28, 92]. The miRNA-146a rs2910164 (G/C) polymorphism is the most widely investigated miRNA SNP related to HNC. A total of 20 comparisons from 18 included studies reported the association between this SNP and HNC risk. Unexpectedly, no significant association was found between this SNP and HNC susceptibility in the overall results of our meta-analysis. In contrast to a previous meta-analysis which found that miR-146a rs2910164 gene polymorphism contributed to HNC risk in Asian populations [91], our meta-analysis did not show any correlation when subgroup analyses among ethnic backgrounds were performed. In regards to cancer types, only increased NPC risk associated with this SNP was found in several comparative models (G vs. C, GG + GC vs. CC and GG vs. CC), suggesting that C allele or CC genotype represented NPC risk factors. However, the conclusion above needs further confirmation since the sample size of this positive pooled result is small (393 cases and 373 controls).

MiR-196a2 is composed of two different mature miRNAs, such as miR-196a-5P and miR-196a-3P, both processed from the same stem-loop [93]. Rs11614913 is located in the mature sequence of miR-196a-3P, thus, a change from C allele to T allele could lead to less efficient processing of the miRNA precursor to its mature form, as well as diminished capacity to regulate target genes [93]. Our systematic review highlighted a significant association between miR-196a2 rs11614913 T allele and increased HNC risk in the co-dominant models. Subgroup analysis results demonstrated that this SNP contributed to OSCC susceptibility, as well as total HNC risk in Asian populations. The results above were consistent with the results of a recently published meta-analysis, which also indicated that the miRNA-196a2 rs11614913 (C/T) may play a risk role in the development of HNC [2]. Hence, our meta-analysis confirmed that the detection of miR-196a2 rs11614913 polymorphism could be useful in HNC prediction and prevention.

SNPs resident in miRNAs gene loci could not only increase cancer risk, but also play protective roles in cancer development. In the present work, we found four miRNA related SNPs, such as miR-499a rs3746444 (T/C), miR-608 rs4919510 (G/C), let-7 rs10877887 (T/C), and miR-124-1 rs531564 (C/G), significantly associated with decreased HNC risk after meta-analysis.

The correlation between SNP rs3746444 in miR-499a and HNC susceptibility was broadly investigated, thus a total of 9 studies were included into our meta-analysis. Although the overall pooled results were not statistically significant, subgroup analysis demonstrated that this SNP was associated with decreased HNC risk, as well as ESCC risk, in Caucasian populations. The rs4919510 is located in the miR-608 mature sequence, thus the mutant C-allelic mir-608 exerts a significantly lower colony formation *in vitro* [64]. In accordance with the functional assay, the overall and subgroup pooled results of the present meta-analysis also indicated that the G > C variation of miR-608 rs4919510 was protective against HNC development.

Let-7 is functionally considered as a tumour suppressor gene due to its role of repressing RAS family [94, 95]. Similarly, miR-124-1 rs531564 GG genotype may promote miR-124 expression, which can directly suppress the expression of its targeting oncogene PTBP1, thus acting as a tumour suppressor [57, 96]. Accordingly, our meta-analysis suggested that the SNPs located in let-7 and miR-124 were both associated with decreased HNC risk.

On the other hand, the overall pooled result demonstrated that miR-34b/c rs4938723 (T/C) was a risk factor for HNC in the co-dominant model, but showed a protective effect on ESCC development in the recessive model. The reason for this divergence is difficult to understand. On the basis of our results we could only postulate that the same miRNA SNP could exert different effects in different cancer types.

Taken together, our present meta-analysis highlighted that systematic and comprehensive literature searching and inclusion, large sample size inclusion, consistency with HWE in almost all of the included cohorts, and reliable meta-analysis results confirmed by sensitive analysis and subgroup analysis, represent important and adequate elements to create a more comprehensive and reliable framework for understanding the potential roles of ncRNA polymorphisms in determining HNC susceptibility. However, there were still some limitations in this systematic review. Firstly, we included oesophageal cancer despite our intention to analyse HNC risk. Only cancers originating from the cervical oesophagus, which begins at the level of cricopharyngeus muscle and extends down to the sternal notch, could be anatomically considered as a HNC subtype. However, after full text screening, we did not find any study investigating ESCC related ncRNA SNPs related to each anatomical cancer location, such as cervical oesophagus, thoracic oesophagus, and abdominal oesophagus. After discussion, we decided to include these studies with the objective to provide a wider perspective for understanding HNC. Secondly, statistical heterogeneity was significant in some meta-analyses, which might be due to the difference between studies in cancer types and ethnic backgrounds. Thirdly, the present meta-analysis was based on unadjusted OR estimates. Lack of sufficient information made a more precise evaluation with ORs adjusted by other covariates including age, gender, smoking status, viral infections, difficult. Fourthly, since quite a few meta-analyses were performed, multiple comparison problem should be considered carefully. Multiple comparison problem may occur when several statistical interferences were considered simultaneously. In this systematic review, five genetic models were used, it may increase the risk of incorrect rejections of null hypotheses, i.e. false positive or type I error. If only one of the five genetic models showed positive result, such as miR-34b/c rs4938723, miR-196a2 rs11614913 and miR-608 rs4919510, the reliability of the result should be considered. Finally, the methodological quality assessed by NOS of several included studies was considered low, which indicated high risk of bias. Inadequate methodological design frequently occurred in the selection of controls. Controls should be frequency-matched in confounding factors, including age, gender and ethnic backgrounds with cases. Rather than just reporting no difference between case and control, the consistency of baseline should be ahead of the selection of controls, or adjust the OR for the confounding factors. Besides, the response rate of case and control was barely reported, and several studies were hospital-based, which could both lead to an increased risk of selection bias. Despite these limitations, our work revealed that an updated and comprehensive meta-analysis is of utmost importance for obtaining reliable results.

## MATERIALS AND METHODS

The protocol of the present systematic review was approved by the institutional review board. Study selection, data extraction and quality assessment were performed by two authors (Pan W and Wu C) in duplicate, and discrepancies were resolved through consensus discussion.

### Inclusion criteria

Studies that met the following inclusion criteria were included in the present systematic review: (1) cohort study or case-control study as study design; (2) patients with diagnosed HNC; (3) controls without any cancer history; (4) study that evaluated the association between ncRNAs polymorphisms and HNC susceptibility; and (5) collect an amount of sufficient data to calculate odds ratios (OR) and 95% confidence intervals (95% CI). Furthermore, patients risk suffering a secondary primary cancer, which might be the result of field cancerization. Based on this, malignancies located in oral cavity, pharynx, larynx, cervical oesophagus, paranasal sinuses, nasal cavity, salivary glands and thyroid gland, were preferred to be considered together. Thus, in the present systematic review, the malignancies mentioned above were regarded as HNC and included.

### Search strategy

The following electronic databases were employed: MEDLINE (via OVID, 1948 to March 16, 2016), EMBASE (via OVID, 1984 to March 16, 2016), Chinese BioMedical Literature Database (CBM, 1978 to March 16, 2016), and China National Knowledge Infrastructure (CNKI, 1994 to March 16, 2016). The search strategy we used combined Medical Subject Headings (MeSH) with free text words. The MeSH terms used were “RNA, Untranslated”, “Polymorphism, Genetic” and “Head and Neck Neoplasms”. Relevant journals and reference lists of included studies were manually searched. The titles and abstracts of all studies were initially scanned to find any eligible study. The full text of the potential eligible studies was obtained to confirm their eligibility.

### Data extraction and quality assessment

For each included study, the following information was extracted: 1) first author name and publication year; 2) country of origin; 3) ethnicity, such as Asian, Caucasian and Black; 4) study design; 5) cancer type; 6) cases and controls number; 7) important information regarding the recruiting process; 8) candidate ncRNAs, with the ancestral allele of a certain genetic loci; and 9) genotype distribution of cases and controls. The data of each cohort were extracted separately and the cohorts were considered as different comparisons in further statistical analyses when more than one cohort was reported in one single study, including cohorts from different countries and cohorts from different regions of one country. Furthermore, since the minor allele frequency in control group varied in different ethnicity background, we decided to take the ancestral allele found in the dbSNP database (available at: http://www.ncbi.nlm.nih.gov/projects/SNP) as the original allele, and the variant alleles at the same genetic locus as exposure factor.

NOS (available at: http://www.ohri.ca/programs/clinical_epidemiology/oxford.asp) was chosen as the tool to assess the methodological quality of included studies. Studies rated 0–3 stars would be considered as high risk of bias, while 4–6 stars 7–9 stars and would be considered as medium and low risk of bias.

### Statistical analysis

Hardy-Weinberg equilibrium (HWE) was used to assess genotype distribution of controls and a goodness-of-fit test (chi-square or Fisher exact test) was performed assess the departure from HWE.

The meta-analysis was performed using RevMan version 5.3 from Cochrane Collaboration, and pooled ORs with 95% CIs were calculated separately in different genetic models to evaluate the association between ncRNA polymorphisms and HNC risk. Using SNP C>T as an example, OR and 95% CI were calculated in four genetic models, including allele contrast (C vs. T), dominant model (CC vs. CT + TT), recessive model (CC + CT vs. TT), and co-dominant model (CC vs. CT, CC vs. TT). The significance of the pooled OR was determined by the 2-tailed z-test and *P* < 0.05 was considered statistically significant. Subgroup analysis was also performed based on ethnicity background and cancer type.

Statistical heterogeneity between studies was analysed by Cochran’s Q statistic following a chi-square distribution and I^2^ statistics. When *P* > 0.05 and I^2^ < 50%, a fixed-effects model was applied; when *p* ≤ 0.05 or I^2^ ≥ 50%, a random-effects model was applied. To ensure the reliability of the results, sensitivity analysis was performed by omitting each study in turn. Publication bias was detected by the funnel plot when the number of included studies was more than 10, since limited number of included studies would affect the power of the tests to distinguish chance from real asymmetry. Harbord test was chosen as test for funnel plot asymmetry. The trim and fill method was applied to identify and correct for funnel plot asymmetry arising from publication bias. Sensitive analysis and detection of publication bias were conducted with STATA Version 14.0 software (Stata Corp, College Station, TX, USA).

## SUPPLEMENTARY MATERIALS FIGURES AND TABLES











## Abbreviations

SNP: single nucleotide polymorphisms
ncRNA: non-coding RNAs
HNC: head and neck cancer
miRNAs: micro RNAs
lncRNAs: long non-coding RNAs
HNSCC: head and neck squamous cell carcinoma
NPC: nasopharyngeal carcinoma
OSCC: oral squamous cell carcinoma
ESCC: oesophageal squamous cell carcinoma
PTC: papillary thyroid carcinoma
LSCC: laryngeal squamous cell carcinoma
TC: thyroid carcinoma
GWAS: Genome-wide association studies
pri-miRNAs: primary miRNAs
pre-miRNAs: precursor miRNAs
UTR: untranslated region
OR: odds ratios
95% CI: 95% confidence intervals
MeSH: Medical Subject Headings
HWE: Hardy-Weinberg equilibrium
NOS: Newcastle–Ottawa quality assessment scale
