# Supplementary material for: Genetic polymorphisms of non-coding RNAs associated with increased head and neck cancer susceptibility: a systematic review and meta-analysis
Source: Oncotarget. 2017 Aug 9;8(37):62508–23. doi: 10.18632/oncotarget.20096 (PMC5617525; doi:10.18632/oncotarget.20096)
Supplement: Supplementary file 1 [file oncotarget-08-62508-s001.pdf]

Genetic polymorphisms of non-coding RNAs associated with increased head and neck cancer susceptibility: a systematic review and meta-analysis

SUPPLEMENTARY MATERIALS

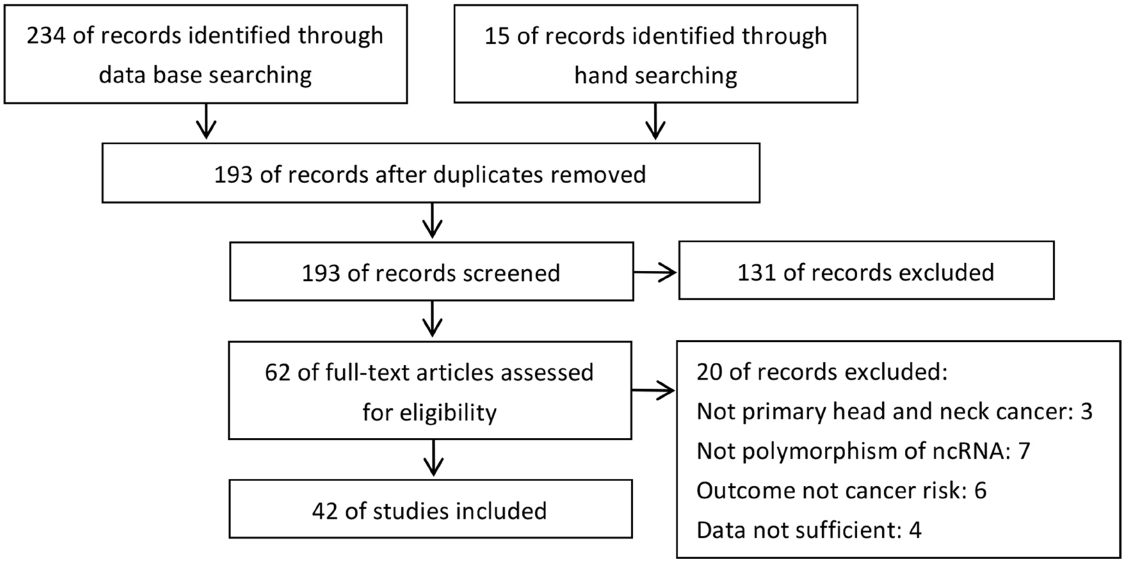

Supplementary Figure 1: Flow diagram of studies identification.

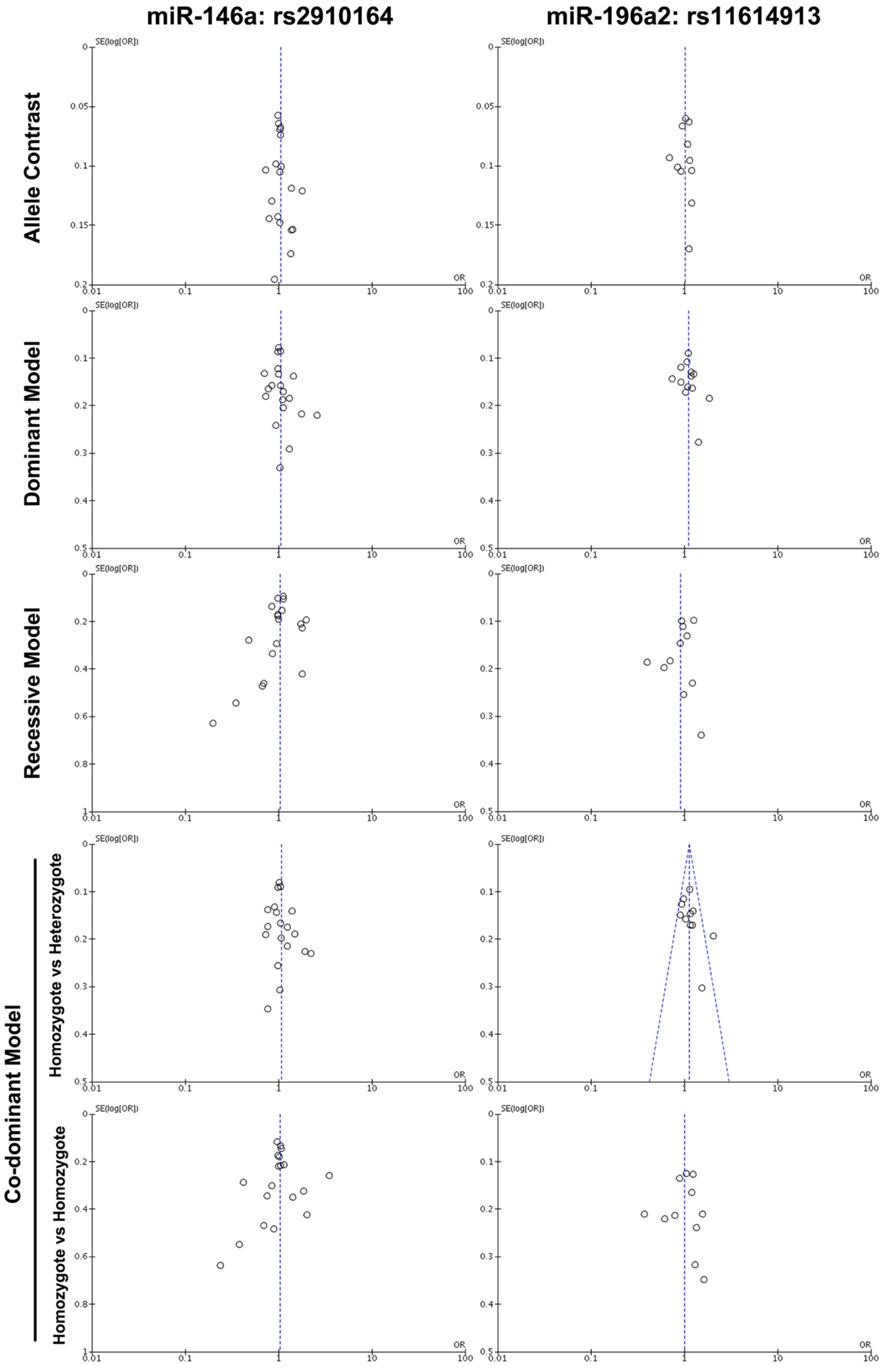

Supplementary Figure 2: Funnel plot of five genetic models of miR-146a rs2910164 and miR-196a2 rs11614913.

Supplementary Table 1: Characteristics of included studies. See Supplementary_Table_1

Supplementary Table 2: Characteristics of SNPs that not eligible for meta-analysis. See Supplementary_Table_2

Supplementary Table 3: Publication bias tested by harbord test

SNPs	Allele Contrast	Dominant Model	Recessive Model	Co-dominant Model	
				Homozygote vs Heterozygote	Homozygote vs Homozygote
**miR-146a rs2910164**	$P = 0.388$	$P = 0.309$	$P = 0.304$	$P = 0.371$	$P = 0.640$
**miR-196a2 rs11614913**	$P = 0.942$	$P = 0.373$	$P = 0.423$	$P = 0.129$	$P = 0.959$

SNP: single nucleotide polymorphisms.

Supplementary Table 4: Overall result of meta-analysis of eligible SNPs. See Supplementary_Table_4

Supplementary Table 5: Subgroup analysis of eligible SNPs. See Supplementary_Table_5
