# Supplementary material for: Genetic polymorphisms of non-coding RNAs associated with increased head and neck cancer susceptibility: a systematic review and meta-analysis
Source: Oncotarget. 2017 Aug 9;8(37):62508–23. doi: 10.18632/oncotarget.20096 (PMC5617525; doi:10.18632/oncotarget.20096)
Supplement: Supplementary file 2 [file oncotarget-08-62508-s002.doc]

**Supplementary Table 1**: Characteristics of Included Studies

| Investigator | Country | Ethnicity | Cancer type | Study design | Genotyping methods | Number  (case/control) | Genotype  (Ancestral) | Other characteristics of the cases/controls | Risk of Bias assessed by NOS | | | |
| --- | --- | --- | --- | --- | --- | --- | --- | --- | --- | --- | --- | --- |
|  |  |  |  |  |  |  |  |  | Selection | Comparability | Exposure | Total |
| Jazdzewski K 2008 [28] | Finland | Caucasian | PTC | CC | SNuPE assay | 206/274 | miR-146a: rs2910164 | Controls were randomly selected from the same region as the cases | **🟑🟑** | — | **🟑🟑** | 4 |
|  | Poland | Caucasian | PTC | CC | SNuPE assay | 201/475 | miR-146a: rs2910164 | Controls were randomly selected from the same region as the cases | **🟑🟑** | — | **🟑🟑** | 4 |
|  | US | Caucasian | PTC | CC | SNuPE assay | 201/152 | miR-146a: rs2910164 | Controls were randomly selected from the same region as the cases | **🟑🟑** | — | **🟑🟑** | 4 |
| Christensen BC 2010 [29] | US | Mixed | HNSCC | CC | TaqMan | 484/555 | miR-196a2: rs11614913 | Controls were randomly selected from the same region as the cases | **🟑🟑🟑** | **🟑🟑** | **🟑🟑** | 7 |
| Guo H 2010 [30] | China | Asian | ESCC | CC | SNuPE assay | 444/468 | miR-146a: rs2910164 | Controls were recruited from routine physical examination | **🟑🟑🟑🟑** | **🟑🟑** | **🟑🟑** | 8 |
| Liu Z 2010 [31] | US | Caucasian | HNSCC | CC | PCR-RFLP | 1109/1130 | miR-146a: rs2910164  miR-149: rs2292832  miR-196a2: rs11614913  miR-499a: rs3746444 | Controls were recruited from healthy visitors | **🟑🟑🟑** | **🟑🟑** | **🟑🟑** | 7 |
| Wang K 2010 [32] | China | Asain | ESCC | CC | SNaPshot | 458/489 | miR-196a2: rs11614913 | Controls were recruited from routine physical examination | **🟑🟑🟑** | **🟑🟑** | **🟑🟑** | 7 |
| Chu Y 2012 [33] | China | Asian | OSCC | CC | PCR-RFLP | 470/425 | miR-146a: rs2910164  miR-149: rs2292832  miR196a2: rs11614913  miR-499a: rs3746444 | All participants were male  Controls were recruited from physical examination  The genotype distribution of miR-149 rs2292832 in control group deviated from HWE (p<0.01) | **🟑🟑🟑🟑** | — | **🟑🟑** | 6 |
| Jones AM 2012 [34] | UK | Caucasian | Non-medullary thyroid cancer | CC | KASPar | 781/6120 | miR-146a: rs2910164  CCAT2:rs6983267  PTCSC2: rs965513 | Controls were recruited from a blood donor service, the 1958 Birth Cohort and another cancer genetic susceptibility study  Characteristics of cases and controls was not provided | **🟑** | — | **🟑** | 2 |
| Tu H 2012 [35] | China | Asian | HNSCC | CC | Sequencing | 273/122 | miR-149: rs2292832 | Controls were recruited from for benign oral lesions, trauma management, pre-prosthetic surgery and removal of impacted teeth. | **🟑🟑** | — | **🟑🟑** | 4 |
| Li L 2013 [36] | China | Asian | NPC | CC | PCR-RFLP | 217/360 | miR-34b/c: rs4938723 | Controls were selected from the same region as the cases | **🟑🟑🟑** | **🟑🟑** | **🟑🟑** | 7 |
| Liu C 2013 [37] | China | Asian | OSCC | CC | PCR-RFLP | 315/92 | miR196a2: rs11614913 | The recruiting process of controls was not clearly described | **🟑** | **🟑🟑** | **🟑🟑** | 5 |
| Lung RW 2013 [38] | China | Asian | NPC | CC | Tm-shift | 233/173 | miR-146a: rs2910164 | Cases and controls were randomly selected from a blood transfusion service | **🟑🟑** | **🟑🟑** | **🟑🟑** | 6 |
| Marino M 2013 [39] | Italy | Caucasian | PTC | CC | Sequencing | 307/206 | miR-146a: rs2910164 | Controls were recruited from healthy volunteers | **🟑🟑🟑** | — | **🟑🟑** | 5 |
| Orsos Z 2013 [40] | Hungary | Caucasian | HNSCC | CC | PCR-CTPP | 468/468 | miR-146a: rs2910164 | Controls were recruited from screening test | **🟑🟑🟑** | **🟑🟑** | **🟑🟑** | 7 |
| Song X 2013 [41] | US | Caucasian | OSCC | CC | PCR-RFLP | 325/335 | miR-146a: rs2910164  miR-149: rs2292832  miR-196a2: rs11614913  miR-499a: rs3746444 | Controls were recruited from healthy visitors of hospital | **🟑🟑🟑🟑** | **🟑🟑** | **🟑🟑** | 8 |
| Umar M 2013 [42] | India | Asian | ESCC | CC | PCR-RFLP  ARMS | 289/308 | miR-146a: rs2910164  miR-196a2: rs11614913  miR-499a: rs3746444  miR-423: rs6505162 | Controls were recruited from blood/organ donors and routine physical examination | **🟑🟑🟑🟑** | **🟑🟑** | **🟑🟑** | 8 |
| Wang Y 2013 [43] | South Africa | Black | ESCC | CC | TaqMan | 368/583 | miR-26a-1: rs7372209  miR-219-1: rs213210  miR-423: rs6505162 | Controls were randomly selected from the same region as the cases  The differences of gender, age, smoking between cases and controls were significant  The genotype distribution of miR-219-1 rs213210 in control group deviated from HWE (p<0.01) | **🟑🟑🟑** | — | **🟑🟑** | 5 |
|  | South Africa | Mixed | ESCC | CC | TaqMan | 197/420 | miR-26a-1: rs7372209  miR-219-1: rs213210  miR-423: rs6505162 | Controls were randomly selected from the same region as the cases  The differences of gender, age, smoking between cases and controls were significant  The genotype distribution of miR-26a-1: rs7372209 and miR-219-1 rs213210 in control group deviated from HWE (p<0.05) | **🟑🟑🟑** | — | **🟑🟑** | 5 |
| Wei J 2013 [44] | China | Asian | ESCC | CC | MALDI-TOF  SNPscan | 380/380 | miR-146a: rs2910164  miR-196a2: rs11614913  miR-26a-1: rs7372209  miR-27a: rs895819  miR-499a: rs3746444 | Controls were recruited from trauma patients  The genotype distribution of miR-26a-1: rs7372209 and miR-499a: rs3746444 in control group deviated from HWE (p<0.05) | **🟑🟑🟑** | **🟑🟑** | **🟑🟑** | 7 |
| Wei W 2013 [45] | China | Asian | PTC | CC | MALDI-TOF | 753/760 | miR-146a: rs2910164 | Controls were recruited from a prospective longitudinal study | **🟑🟑🟑🟑** | **🟑🟑** | **🟑🟑** | 8 |
| Wu H 2013 [46] | China | Asian | ESCC | CC | MALDI-TOF | 748/771 | ENST00000506071: rs3756087  ENST00000510727: rs6051321  ENST00000510727: rs13038142  ENST00000510727: rs6084145  NR_002319: rs12768993  NR_002319: rs7489  NR_002319: rs829225  NR_002319: rs11815169  NR_002319: rs12571819  NR_002319: rs12570608  NR_024015: rs2304285  NR_024015: rs8506  NR_024015: rs9312  NR_027266: rs34230967  NR_027266: rs6517211  NR_033415: rs17029673  NR_033415: rs17841343  NR_033415: rs7405662  NR_033415: rs11657092  NR_033415: rs4494601  NR_033415: rs34119367  NR_033415: rs76652990  NR_033415: rs8076409  NR_033415: rs8070585  NR_033415: rs6416918  NR_033844: rs5028631  POLR2E: rs3787016  PTCSC2: rs965513  uc002krz.2: rs9945350  uc002krz.2: rs9945589  uc002krz.2: rs9945456  uc002vga.1: rs207893  uc002vga.1: rs207894  uc002vga.1: rs207895  uc002vgl.2: rs1179724  uc002yug.2: rs2246640  uc002yug.2: rs2070369  uc003frr.1: rs6777331  uc003frr.1: rs13080878  uc003frr.1: rs2292893  uc003frr.1: rs17806488  uc003frr.1: rs17744708  uc003frr.1: rs67435657  uc003frr.1: rs35404821  uc003opf.1: rs11752942  uc003opf.1: rs2477757  uc003opf.1: rs4711631  uc003opf.1: rs4714336  uc003opf.1: rs11752896  uc003opf.1: rs13203076  uc003opf.1: rs16893397  uc010djj.1: rs2760741  uc010djj.1: rs2760740  uc010djj.1: rs2957926 | Controls were recruited from a nutritional survey | **🟑🟑🟑🟑** | **🟑🟑** | **🟑🟑🟑** | 9 |
|  | China | Asain | ESCC | CC | MALDI-TOF | 745/782 | uc003opf.1: rs11752942 | Controls were recruited from a community-based screen program | **🟑🟑🟑🟑** | **🟑🟑** | **🟑🟑** | 8 |
| Yin J 2013 [47] | China | Asian | ESCC | CC | PCR-LDR | 629/686 | miR-34b/c: rs4938723  miR-124-1: rs531564  miR-125a: rs12975333  miR-423: rs6505162 | Controls were recruited from trauma patients | **🟑🟑🟑** | **🟑🟑** | **🟑🟑** | 7 |
| Huang G 2014 [48] | China | Asian | NPC | CC | PCR-RFLP | 160/200 | miR-146a: rs2910164 | Controls were recruited from routine physical examination | **🟑🟑🟑🟑** | **—** | **🟑🟑** | 6 |
| Jiang L 2014 [49] | China | Asian | ESCC | CC | PCR-LDR | 706/745 | miR-218-2: rs11134527 | Controls were recruited from the same hospital as the cases | **🟑🟑** | — | **🟑🟑** | 4 |
| Li P 2014 [50] | China | Asian | NPC | CC | TaqMan | 1020/1006 | miR196a2: rs11614913 | Controls were recruited from a community cancer screen program | **🟑🟑🟑** | **🟑🟑** | **🟑🟑** | 7 |
| Lin D 2014 [51] | China | Asian | Laryngeal Carcinoma | CC | TaqMan | 204/440 | miR-146a: rs2910164 | Controls were recruited from routine physical examination | **🟑🟑🟑🟑** | **🟑🟑** | **🟑🟑** | 8 |
| Palmieri A 2014 [52] | Italy | Caucasian | OSCC | CC | TaqMan | 337/88 | miR-146a: rs2910164 | Controls were recruited from a public database  Characteristics of cases and controls was not provided | **🟑🟑🟑** | — | **🟑** | 4 |
| Qu Y 2014 [53] | China | Asian | ESCC | CC | AS-PCR | 381/426 | miR-146a: rs2910164  miR-196a2: rs11614913 | Controls were recruited from a digestive disease census | **🟑🟑🟑🟑** | **🟑🟑** | **🟑🟑** | 8 |
| Roy R 2014 [54] | India | Asian | OSCC | CC | TaqMan | 451/452 | miR-34b: rs2187473  miR-196a2: rs11614913 | All participants reported tobacco habits  Controls were recruited from patients with dental ailments | **🟑** | — | **🟑🟑** | 3 |
| Wang N 2014 [55] | China | Asian | ESCC | CC | PCR-LDR | 597/597 | MiR-196a2: rs11614913 | All participants were recruited from a endoscopic screening program | **🟑🟑🟑🟑** | **🟑🟑** | **🟑🟑** | 8 |
| Wei W 2014 [56] | China | Asian | PTC | CC | MALDI-TOF | 838/1006 | miR-149: rs2292832 | Controls were recruited from a prospective longitudinal study | **🟑🟑🟑🟑** | — | **🟑🟑** | 6 |
| Zhang J 2014 [57] | China | Asian | ESCC | CC | SNuPE assay | 1109/1275 | miR-26a-1: rs7372209  miR-27a: rs895819  miR-34b/c: rs4938723  miR-124-1: rs531564  miR-218-2: rs11134527 | Controls were recruited from a prospective longitudinal study. | **🟑🟑🟑🟑** | **🟑🟑** | **🟑🟑** | 8 |
| Zhang X 2014 [58] | China | Asian | ESCC | CC | PCR-RFLP | 1000/1000 | HOTAIR: rs920778  HOTAIR: rs1899663  HOTAIR: rs4759314 | Controls were recruited from a community-based screening program | **🟑🟑🟑** | **🟑🟑** | **🟑🟑** | 7 |
|  | China | Asian | ESCC | CC | PCR-RFLP | 510/550 | HOTAIR: rs920778 | Controls were recruited from routine physical examination | **🟑🟑🟑🟑** | **🟑🟑** | **🟑🟑** | 8 |
|  | China | Asian | ESCC | CC | PCR-RFLP | 588/600 | HOTAIR: rs920778 | Controls were recruited from a community-based screening program | **🟑🟑🟑🟑** | **🟑🟑** | **🟑🟑** | 8 |
| Zhang Y 2014 [59] | China | Asian | OSCC | CC | TaqMan | 384/731 | let-7: rs10877887  let-7: rs13293512 | Controls were recruited from a community-based screening program | **🟑🟑🟑🟑** | **🟑🟑** | **🟑🟑** | 8 |
| Buas MF 2015 [60] | US | Caucasian | Esophageal Adenocarcinoma | CC | Genotyping | 2515/3207 | miR-487a: rs7342570  miR-638: rs12232826  miR-3117: rs7526812  miR-3188: rs12461701  miR-3612: rs1709696  miR-4421: rs12564376  miR-4467: rs12534337  miR-4513: rs1378940  miR-4521: rs7210250  miR-4725: rs17880825  miR-4756: rs3787547  miR-5186: rs9842591  miR-5579: rs10899620 | Controls were recruited from cancer genetic susceptibility studies | **🟑🟑🟑** | — | **🟑🟑** | 5 |
| Chen P 2015 [61] | China | Asian | PTC | CC | PCR-RFLP | 784/1006 | miR-34b/c: rs4938723 | Controls were recruited from routine physical examination | **🟑🟑🟑🟑** | — | **🟑🟑** | 6 |
| Hou Y 2015 [62] | China | Asian | OSCC | CC | TaqMan | 155/204 | miR-499a: rs3746444 | All participants were male, and habitually chewed betel quid  Controls were recruited from health screen campaigns | **🟑🟑🟑🟑** | **🟑🟑** | **🟑🟑** | 8 |
|  | China | Asian | OSCC | CC | TaqMan | 512/668 | miR-499a: rs3746444 | Controls were non-cancer patients recruited from other hospital departments, such as Orthopedics | **🟑🟑🟑** | **🟑🟑** | **🟑🟑** | 7 |
| Kang M 2015 [63] | China | Asian | ESCC | CC | MALDI-TOF | 380/380 | ANRIL: rs2151280  HULC: rs7763881  POLR2E: rs3787016 | Controls were recruited from trauma and infectious patients | **🟑🟑🟑** | — | **🟑🟑** | 5 |
| Qiu F 2015 [64] | China | Asian | NPC | CC | TaqMan | 906/1072 | miR-499a: rs3746444  miR-608: rs4919510  miR-3152: rs13299349  miR-4293: rs12220909  miR-4513: rs2168518  miR-4520a: rs8078913  miR-5579: rs11237828  miR-5689: rs9295535 | Controls were randomly selected from healthy check-up programs | **🟑🟑🟑🟑** | **🟑🟑** | **🟑🟑** | 8 |
|  | China | Asian | NPC | CC | TaqMan | 684/907 | miR-608: rs4919510 | Controls were randomly selected from a nutritional survey | **🟑🟑🟑** | **🟑🟑** | **🟑🟑** | 7 |
| Song X 2015 [65] | China | Asian | ESCC | CC | MALDI-TOF | 248/300 | miR-219-1: rs107822  miR-219-1: rs213210 | Controls were recruited from healthy visitors of outpatient clinic  The genotype distribution of miR-219-1 rs107822 in control group deviated from HWE (p<0.01) | **🟑🟑🟑🟑** | **🟑🟑** | **🟑🟑** | 8 |
| Wang Y 2015 [66] | China | Asian | PTC | CC | PCR-RFLP  TaqMAN | 618/562 | let-7: rs10877887  let-7: rs13293512 | Controls were recruited from routine physical examination  The genotype distribution of let-7: rs13293512 in control group deviated from HWE (p<0.05) | **🟑🟑🟑🟑** | **🟑🟑** | **🟑🟑** | 8 |
| Wei W 2015 [67] | China | Asian | PTC | CC | MALDI-TOF | 828/1038 | miR-146a: rs2910164  miR-196a2: rs11614913  miR-449b: rs10061133  miR-499a: rs3746444  miR-608: rs4919510  miR-627: rs2620381  miR-646: rs6513497  miR-933: rs79402775  miR-1269: rs73239138  miR-3144: rs67106263  miR-3152: rs13299349  miR-4293: rs12220909 | Controls were recruited from a prospective longitudinal study | **🟑🟑🟑🟑** | — | **🟑🟑** | 6 |
| Zhang P 2015 [68] | China | Asian | ESCC | CC | SNuPE assay | 773/882 | miR-449b: rs10061133  miR-608: rs4919510  miR-627: rs2620381  miR-646: rs6513497  miR-4293: rs12220909 | Controls were recruited from a prospective longitudinal study. | **🟑🟑🟑🟑** | **🟑🟑** | **🟑🟑** | 8 |
| Zhang X 2015 [69] | China | Asian | PTC | CC | MALDI-TOF | 1238/1275 | miR-146a: rs2910164 | Controls were selected from a routine health examination and a survey of chronic disease | **🟑🟑🟑** | — | **🟑🟑** | 6 |

SNPs: single nucleotide polymorphisms; NOS: Newcastle–Ottawa quality assessment scale; ESCC: esophageal squamous cell carcinoma ;HNSCC: head and neck squamous cell carcinoma; NPC: nasopharyngeal carcinoma ; OSCC: oral squamous cell carcinoma; PTC: papillary thyroid carcinoma; CC: case control; SNuPE assay: single nucleotide primer extension assay; PCR-RFLP: polymerase chain reaction-restriction fragment length polymorphism; PCR-CTPP: polymerase chain reaction with confronting two-pair primers; ARMS: amplification refractory mutation system; MALDI-TOF: matrix-assisted laser desorption/ionization time of flight mass spectrometry assay; PCR-LDR: polymerase chain reaction-ligation detection reaction; AS-PCR: allele specific polymerase chain reaction.
