# Supplementary material for: Genetic polymorphisms of non-coding RNAs associated with increased head and neck cancer susceptibility: a systematic review and meta-analysis
Source: Oncotarget. 2017 Aug 9;8(37):62508–23. doi: 10.18632/oncotarget.20096 (PMC5617525; doi:10.18632/oncotarget.20096)
Supplement: Supplementary file 3 [file oncotarget-08-62508-s003.doc]

**Supplementary Table 2:** Characteristics of SNPs that not eligible for meta-analysis

| ncRNAs | SNPs | SNP Alleles | Ethnic | Cancer Type | Reference |
| --- | --- | --- | --- | --- | --- |
| lncRNAs | | | | | |
| ANRIL | rs2151280 | C/T | Asian | ESCC | 63 |
| CCAT2 | rs6983267 | G/T | Caucasian | TC | 34 |
| ENST00000506071 | rs3756087 | A/G | Asian | ESCC | 46 |
| ENST00000510727 | rs6051321 | T/C | Asian | ESCC | 46 |
| ENST00000510727 | rs13038142 | G/A | Asian | ESCC | 46 |
| ENST00000510727 | rs6084145 | T/C | Asian | ESCC | 46 |
| HOTAIR | rs1899663 | G/T | Asian | ESCC | 58 |
| HOTAIR | rs4759314 | G/A | Asian | ESCC | 58 |
| HULC | rs7763881 | A/C | Asian | ESCC | 63 |
| NR_002319 | rs12768993 | G/A | Asian | ESCC | 46 |
| NR_002319 | rs7489 | G/A | Asian | ESCC | 46 |
| NR_002319 | rs829225 | A/G | Asian | ESCC | 46 |
| NR_002319 | rs11815169 | T/C | Asian | ESCC | 46 |
| NR_002319 | rs12571819 | T/C | Asian | ESCC | 46 |
| NR_002319 | rs12570608 | C/T | Asian | ESCC | 46 |
| NR_024015 | rs2304285 | G/A | Asian | ESCC | 46 |
| NR_024015 | rs8506 | C/T | Asian | ESCC | 46 |
| NR_024015 | rs9312 | C/T | Asian | ESCC | 46 |
| NR_027266 | rs34230967 | C/T | Asian | ESCC | 46 |
| NR_027266 | rs6517211 | G/A | Asian | ESCC | 46 |
| NR_033415 | rs17029673 | C/T | Asian | ESCC | 46 |
| NR_033415 | rs17841343 | A/G | Asian | ESCC | 46 |
| NR_033415 | rs7405662 | A/G | Asian | ESCC | 46 |
| NR_033415 | rs11657092 | C/T | Asian | ESCC | 46 |
| NR_033415 | rs4494601 | A/G | Asian | ESCC | 46 |
| NR_033415 | rs34119367 | A/G | Asian | ESCC | 46 |
| NR_033415 | rs76652990 | C/G | Asian | ESCC | 46 |
| NR_033415 | rs8076409 | C/G | Asian | ESCC | 46 |
| NR_033415 | rs8070585 | C/T | Asian | ESCC | 46 |
| NR_033415 | rs6416918 | C/T | Asian | ESCC | 46 |
| NR_033844 | rs5028631 | A/G | Asian | ESCC | 46 |
| POLR2E | rs3787016 | A/G | Asian | ESCC | 63 |
| PTCSC2 | rs965513 | A/G | Caucasian | TC | 34 |
| uc002krz.2 | rs9945350 | C/T | Asian | ESCC | 46 |
| uc002krz.2 | rs9945589 | A/G | Asian | ESCC | 46 |
| uc002krz.2 | rs9945456 | A/G | Asian | ESCC | 46 |
| uc002vga.1 | rs207893 | A/T | Asian | ESCC | 46 |
| uc002vga.1 | rs207894 | A/G | Asian | ESCC | 46 |
| uc002vga.1 | rs207895 | A/G | Asian | ESCC | 46 |
| uc002vgl.2 | rs1179724 | A/G | Asian | ESCC | 46 |
| uc002yug.2 | rs2246640 | C/T | Asian | ESCC | 46 |
| uc002yug.2 | rs2070369 | G/A | Asian | ESCC | 46 |
| uc003frr.1 | rs6777331 | C/T | Asian | ESCC | 46 |
| uc003frr.1 | rs13080878 | A/G | Asian | ESCC | 46 |
| uc003frr.1 | rs2292893 | A/G | Asian | ESCC | 46 |
| uc003frr.1 | rs17806488 | A/G | Asian | ESCC | 46 |
| uc003frr.1 | rs17744708 | C/T | Asian | ESCC | 46 |
| uc003frr.1 | rs67435657 | C/T | Asian | ESCC | 46 |
| uc003frr.1 | rs35404821 | C/T | Asian | ESCC | 46 |
| uc003opf.1 | rs2477757 | C/T | Asian | ESCC | 46 |
| uc003opf.1 | rs4711631 | C/A | Asian | ESCC | 46 |
| uc003opf.1 | rs4714336 | C/T | Asian | ESCC | 46 |
| uc003opf.1 | rs11752896 | A/G | Asian | ESCC | 46 |
| uc003opf.1 | rs13203076 | T/C | Asian | ESCC | 46 |
| uc003opf.1 | rs16893397 | A/G | Asian | ESCC | 46 |
| uc010djj.1 | rs2760741 | A/G | Asian | ESCC | 46 |
| uc010djj.1 | rs2760740 | A/G | Asian | ESCC | 46 |
| uc010djj.1 | rs2957926 | C/T | Asian | ESCC | 46 |
| miRNAs | | | | | |
| miR-34b | rs2187473 | G/A | Asian | OSCC | 54 |
| miR-125a | rs12975333 | G/T | Asian | ESCC | 47 |
| miR-219-1 | rs107822 | C/T | Asian | ESCC | 65 |
| miR-487a | rs7342570 | A/G | Caucasian | EA | 60 |
| miR-638 | rs12232826 | G/T | Caucasian | EA | 60 |
| miR-933 | rs79402775 | G/A | Asian | PTC | 67 |
| miR-1269 | rs73239138 | G/A | Asian | PTC | 67 |
| miR-3117 | rs7526812 | A/G | Caucasian | EA | 60 |
| miR-3144 | rs67106263 | G/A | Asian | PTC | 67 |
| miR-3188 | rs12461701 | C/T | Caucasian | EA | 60 |
| miR-3612 | rs1709696 | A/G/T | Caucasian | EA | 60 |
| miR-4421 | rs12564376 | C/T | Caucasian | EA | 60 |
| miR-4467 | rs12534337 | C/T | Caucasian | EA | 60 |
| miR-4513 | rs1378940 | G/T | Caucasian | EA | 60 |
| miR-4513 | rs2168518 | C/T | Asian | NPC | 64 |
| miR-4520a | rs8078913 | C/T | Asian | NPC | 64 |
| miR-4521 | rs7210250 | A/G | Caucasian | EA | 60 |
| miR-4725 | rs17880825 | C/T | Caucasian | EA | 60 |
| miR-4756 | rs3787547 | A/G | Caucasian | EA | 60 |
| miR-5186 | rs9842591 | A/C | Caucasian | EA | 60 |
| miR-5579 | rs10899620 | A/G | Caucasian | EA | 60 |
| miR-5579 | rs11237828 | T/C | Asian | NPC | 64 |
| miR-5689 | rs9295535 | T/C | Asian | NPC | 64 |

ncRNA: noncoding RNAs; SNPs: single nucleotide polymorphisms; lncRNAs: long noncoding RNAs; miRNAs: micro RNAs; ESCC: esophageal squamous cell carcinoma; TC: thyroid carcinoma; OSCC: oral squamous cell carcinoma; EA: esophageal adenoma; PTC: papillary thyroid carcinoma; NPC: nasopharyngeal carcinoma.
