# Supplementary material for: Genetic polymorphisms of non-coding RNAs associated with increased head and neck cancer susceptibility: a systematic review and meta-analysis
Source: Oncotarget. 2017 Aug 9;8(37):62508–23. doi: 10.18632/oncotarget.20096 (PMC5617525; doi:10.18632/oncotarget.20096)
Supplement: Supplementary file 4 [file oncotarget-08-62508-s004.doc]

**Supplementary Table 4:** Overall result of meta-analysis of eligible SNPs

| **SNPs** | **Allele Contrast** | | | | | **Dominant Model** | | | | | **Recessive Model** | | | | | **Co-dominant Model** | | | | | | | | | |
| --- | --- | --- | --- | --- | --- | --- | --- | --- | --- | --- | --- | --- | --- | --- | --- | --- | --- | --- | --- | --- | --- | --- | --- | --- | --- |
|  |  |  |  |  |  |  |  |  |  |  |  |  |  |  |  | **Homozygote vs Heterozygote** | | | | | **Homozygote vs Homozygote** | | | | |
|  | n | OR [95% CI] | P | P-H | I^2^ | n | OR [95% CI] | P | P-H | I^2^ | n | OR [95% CI] | P | P-H | I^2^ | n | OR [95% CI] | P | P-H | I^2^ | n | OR [95% CI] | P | P-H | I^2^ |
| **HOTAIR:**  **rs920778** | 3 | **1.46**  **[1.32, 1.61]** | <0.01 | 0.37 | 1 | 3 | **1.44**  **[1.27, 1.62]** | <0.01 | 0.40 | 0 | 3 | **2.54**  **[1.93, 3.34]** | <0.01 | 0.37 | 0 | 3 | **1.29**  **[1.14, 1.47]** | <0.01 | 0.43 | 0 | 3 | **2.81**  **[2.13, 3.71]** | <0.01 | 0.36 | 2 |
| **uc003opf.1:**  **rs11752942** | 2 | **0.75**  **[0.68, 0.83]** | <0.01 | 0.57 | 0 | 2 | **0.72**  **[0.62, 0.83]** | <0.01 | 0.50 | 0 | 2 | **0.62**  **[0.50, 0.78]** | <0.01 | 0.91 | 0 | 2 | **0.77**  **[0.66, 0.89]** | <0.01 | 0.53 | 0 | 2 | **0.54**  **[0.43, 0.69]** | <0.01 | 0.74 | 0 |
| **let-7:**  **rs10877887** | 2 | 0.90  [0.79, 1.02] | 0.10 | 0.84 | 0 | 2 | **0.80**  **[0.68, 0.95]** | 0.01 | 0.81 | 0 | 2 | 1.08  [0.82, 1.42] | 0.56 | 0.33 | 0 | 2 | **0.77**  **[0.64, 0.92]** | <0.01 | 0.54 | 0 | 2 | 0.96  [0.72, 1.27] | 0.77 | 0.42 | 0 |
| **let-7:**  **rs13293512** | 2 | 0.90  [0.80, 1.02] | 0.10 | 0.34 | 0 | 2 | 0.88  [0.73, 1.06] | 0.18 | 0.51 | 0 | 2 | 0.86  [0.69, 1.07] | 0.17 | 0.36 | 0 | 2 | 0.91  [0.74, 1.10] | 0.33 | 0.69 | 0 | 2 | 0.80  [0.63, 1.03] | 0.09 | 0.32 | 0 |
| **miR-26a-1:**  **rs7372209** | 4 | 0.81  [0.60, 1.10] | 0.56 | <0.01 | 78 | 4 | 0.75  [0.53, 1.07] | 0.12 | <0.01 | 77 | 3 | 1.22  [0.95, 1.56] | 0.12 | 0.90 | 0 | 4 | 0.74  [0.52, 1.03] | 0.04 | <0.01 | 74 | 3 | 1.19  [0.92, 1.54] | 0.18 | 0.84 | 0 |
| **miR-27a:**  **rs895819** | 2 | 1.00  [0.89, 1.12] | 0.99 | 0.25 | 25 | 2 | 1.02  [0.88, 1.17] | 0.82 | 0.48 | 0 | 2 | 0.88  [0.55, 1.39] | 0.67 | 0.15 | 52 | 2 | 1.03  [0.89, 1.19] | 0.70 | 0.77 | 0 | 2 | 0.88  [0.55, 1.43] | 0.73 | 0.14 | 54 |
| **miR-34b/c:**  **rs4938723** | 4 | 1.13  [0.92, 1.39] | <0.01 | <0.01 | 85 | 4 | 1.23  [0.97, 1.55] | <0.01 | <0.01 | 78 | 4 | 1.04  [0.70, 1.56] | 0.64 | <0.01 | 81 | 4 | **1.23**  **[1.03, 1.47]** | <0.01 | 0.06 | 59 | 4 | 1.17  [0.71, 1.93] | 0.11 | <0.01 | 86 |
| **miR-124-1:**  **rs531564** | 2 | 0.88  [0.78, 1.01] | 0.06 | 0.73 | 0 | 2 | 0.92  [0.80, 1.07] | 0.27 | 0.57 | 0 | 2 | **0.46**  **[0.28, 0.75]** | <0.01 | 0.31 | 1 | 2 | 0.97  [0.84, 1.13] | 0.70 | 0.47 | 0 | 2 | **0.45**  **[0.27, 0.75]** | <0.01 | 0.34 | 0 |
| **miR-146a:**  **rs2910164** | 19 | 1.06  [0.97, 1.15] | 0.20 | <0.01 | 68 | 20 | 1.06  [0.95, 1.18] | 0.29 | <0.01 | 61 | 19 | 1.04  [0.89, 1.20] | 0.65 | <0.01 | 64 | 19 | 1.07  [0.96, 1.20] | 0.24 | <0.01 | 58 | 19 | 1.03  [0.85, 1.24] | 0.77 | <0.01 | 64 |
| **miR-149:**  **rs2292832** | 4 | 0.97  [0.82, 1.15] | 0.72 | 0.02 | 68 | 5 | 0.98  [0.72, 1.32] | 0.88 | <0.01 | 75 | 4 | 0.91  [0.80, 1.04] | 0.17 | 0.45 | 0 | 4 | 0.99  [0.66, 1.48] | 0.97 | <0.01 | 78 | 4 | 0.96  [0.60, 1.52] | 0.86 | <0.01 | 79 |
| **miR-196a2:**  **rs11614913** | 11 | 1.01  [0.92, 1.11] | 0.84 | <0.01 | 69 | 13 | 1.10  [0.99, 1.22] | 0.08 | 0.04 | 46 | 11 | 0.90  [0.74, 1.09] | 0.27 | <0.01 | 76 | 11 | **1.12**  **[1.03, 1.22]** | **0.01** | 0.07 | 42 | 11 | 1.00  [0.79, 1.25] | 0.97 | <0.01 | 76 |
| **miR-218-2:**  **rs11134527** | 2 | 1.06  [0.88, 1.27] | 0.40 | 0.05 | 73 | 2 | 1.12  [0.69, 1.82] | 0.50 | <0.01 | 86 | 2 | 1.05  [0.92, 1.20] | 0.49 | 0.42 | 0 | 2 | 1.11  [0.68, 1.80] | 0.63 | 0.01 | 84 | 2 | 1.14  [0.71, 1.85] | 0.40 | 0.02 | 83 |
| **miR-219-1:**  **rs213210** | 3 | 0.93  [0.76, 1.13] | 0.46 | 0.39 | 0 | 3 | 0.92  [0.74, 1.13] | 0.42 | 0.33 | 9 | 1 | N/A | N/A | N/A | N/A | 3 | 0.91  [0.74, 1.13] | 0.41 | 0.33 | 10 | 1 | N/A | N/A | N/A | N/A |
| **miR-423:**  **rs6505162** | 4 | 1.12  [1.00, 1.26] | 0.05 | 0.25 | 26 | 4 | 1.13  [0.98, 1.31] | 0.09 | 0.46 | 0 | 4 | 1.23  [0.95, 1.61] | 0.12 | 0.17 | 41 | 4 | 1.10  [0.94, 1.27] | 0.23 | 0.66 | 0 | 4 | 1.28  [0.96, 1.71] | 0.09 | 0.13 | 47 |
| **miR-449b:**  **rs10061133** | 2 | 0.92  [0.82, 1.02] | 0.12 | 0.28 | 13 | 2 | 0.93  [0.81, 1.06] | 0.28 | 0.53 | 0 | 2 | 0.79  [0.52, 1.20] | 0.11 | 0.12 | 58 | 2 | 0.96  [0.83, 1.10] | 0.53 | 0.83 | 0 | 2 | 0.77  [0.50, 1.19] | 0.09 | 0.12 | 59 |
| **miR-499a:**  **rs3746444** | 8 | 0.99  [0.83, 1.20] | 0.45 | <0.01 | 81 | 9 | 0.94  [0.78, 1.13] | 0.08 | <0.01 | 78 | 8 | 1.12  [0.90, 1.41] | 0.31 | 0.06 | 48 | 8 | 0.95  [0.78, 1.15] | 0.10 | <0.01 | 75 | 8 | 1.06  [0.71, 1.58] | 0.46 | 0.03 | 56 |
| **miR-608:**  **rs4919510** | 4 | 0.91  [0.80, 1.04] | <0.01 | <0.01 | 75 | 4 | 0.93  [0.79, 1.11] | 0.20 | 0.04 | 64 | 4 | **0.82**  **[0.68, 0.98]** | <0.01 | 0.07 | 58 | 4 | 0.99  [0.89, 1.11] | 0.88 | 0.21 | 33 | 4 | 0.81  [0.63, 1.06] | 0.12 | 0.01 | 72 |
| **miR-627:**  **rs2620381** | 2 | 0.98  [0.84, 1.15] | 0.23 | 0.67 | 0 | 2 | 0.97  [0.82, 1.16] | 0.76 | 0.51 | 0 | 2 | 1.10  [0.56, 2.16] | 0.27 | 0.44 | 0 | 2 | 0.97  [0.81, 1.15] | 0.38 | 0.40 | 0 | 2 | 1.09  [0.55, 2.14] | 0.80 | 0.46 | 0 |
| **miR-646:**  **rs6513497** | 2 | 0.94  [0.80, 1.10] | 0.41 | 0.71 | 0 | 2 | 0.95  [0.81, 1.13] | 0.59 | 0.53 | 0 | 2 | 0.60  [0.29, 1.25] | 0.17 | 0.41 | 0 | 2 | 0.98  [0.82, 1.16] | 0.79 | 0.41 | 0 | 2 | 0.60  [0.29, 1.24] | 0.17 | 0.43 | 0 |
| **miR-3152:**  **rs13299349** | 2 | 1.12  [0.98, 1.28] | 0.10 | 0.74 | 0 | 2 | 1.14  [0.98, 1.32] | 0.09 | 0.76 | 0 | 2 | 1.11  [0.70, 1.78] | 0.65 | 0.88 | 0 | 2 | 1.14  [0.97, 1.33] | 0.10 | 0.78 | 0 | 2 | 1.14  [0.71, 1.83] | 0.57 | 0.87 | 0 |
| **miR-4293:**  **rs12220909** | 3 | 0.94  [0.78, 1.14] | 0.42 | 0.03 | 73 | 3 | 0.92  [0.74, 1.15] | 0.25 | 0.02 | 74 | 3 | 1.07  [0.81, 1.41] | 0.61 | 0.67 | 0 | 3 | 0.91  [0.73, 1.14] | 0.18 | 0.03 | 72 | 3 | 1.06  [0.81, 1.41] | 0.66 | 0.55 | 0 |

SNPs: single nucleotide polymorphisms; n: number of cohorts; OR: odd ratio; CI: confidence interval; P-H: p value of heterogeneity.
