# Supplementary material for: Genetic polymorphisms of non-coding RNAs associated with increased head and neck cancer susceptibility: a systematic review and meta-analysis
Source: Oncotarget. 2017 Aug 9;8(37):62508–23. doi: 10.18632/oncotarget.20096 (PMC5617525; doi:10.18632/oncotarget.20096)
Supplement: Supplementary file 5 [file oncotarget-08-62508-s005.doc]

**Supplementary Table 5:** Subgroup analysis of eligible SNPs

| **SNPs** | **Allele Contrast** | | | | | **Dominant Model** | | | | | **Recessive Model** | | | | | **Co-dominant Model** | | | | | | | | | |
| --- | --- | --- | --- | --- | --- | --- | --- | --- | --- | --- | --- | --- | --- | --- | --- | --- | --- | --- | --- | --- | --- | --- | --- | --- | --- |
|  |  |  |  |  |  |  |  |  |  |  |  |  |  |  |  | **Homozygote vs Heterozygote** | | | | | **Homozygote vs Homozygote** | | | | |
|  | n | OR [95% CI] | P | P-H | I^2^ | n | OR [95% CI] | P | P-H | I^2^ | n | OR [95% CI] | P | P-H | I^2^ | n | OR [95% CI] | P | P-H | I^2^ | n | OR [95% CI] | P | P-H | I^2^ |
| **mir-26a-1: rs7372209 (C/T)** | | | | | |  |  |  |  |  |  |  |  |  |  |  |  |  |  |  |  |  |  |  |  |
| **Over all** | 4 | 0.81  [0.60, 1.10] | 0.56 | <0.01 | 78 | 4 | 0.75  [0.53, 1.07] | 0.12 | <0.01 | 77 | 3 | 1.22  [0.95, 1.56] | 0.12 | 0.90 | 0 | 4 | 0.74  [0.52, 1.03] | 0.04 | <0.01 | 74 | 3 | 1.19  [0.92, 1.54] | 0.18 | 0.84 | 0 |
| **Ethnic** |  |  |  |  |  |  |  |  |  |  |  |  |  |  |  |  |  |  |  |  |  |  |  |  |  |
| Asian | 2 | 1.03  [0.93, 1.15] | 0.54 | 0.55 | 0 | 2 | 0.99  [0.86, 1.14] | 0.90 | 0.50 | 0 | 2 | 1.23  [0.96, 1.57] | 0.11 | 1 | 0 | 2 | 0.95  [0.82, 1.10] | 0.52 | 0.52 | 0 | 2 | 1.20  [0.93, 1.55] | 0.16 | 0.87 | 0 |
| **miR-34b/c: rs4938723 (T/C)** | | | | | |  |  |  |  |  |  |  |  |  |  |  |  |  |  |  |  |  |  |  |  |
| **Over all** | 4 | 1.13  [0.92, 1.39] | 0.23 | <0.01 | 85 | 4 | 1.23  [0.97, 1.55] | 0.09 | <0.01 | 78 | 4 | 1.04  [0.70, 1.56] | 0.84 | 0.01 | 81 | 4 | **1.23**  **[1.03, 1.47]** | 0.02 | 0.06 | 59 | 4 | 1.17  [0.71, 1.93] | 0.54 | <0.01 | 86 |
| **Cancer type** |  |  |  |  |  |  |  |  |  |  |  |  |  |  |  |  |  |  |  |  |  |  |  |  |  |
| ESCC | 2 | 0.97  [0.88,1.07] | 0.54 | 0.48 | 0 | 2 | 1.03  [0.91, 1.18] | 0.64 | 0.70 | 0 | 2 | **0.77**  **[0.60, 0.97]** | 0.03 | 0.38 | 0 | 2 | 1.08  [0.95, 1.24] | 0.25 | 0.92 | 0 | 2 | 0.80  [0.62, 1.02] | 0.07 | 0.38 | 0 |
| **miR-146a: rs2910164 (G/C)** | | | | | | |  |  |  |  |  |  |  |  |  |  |  |  |  |  |  |  |  |  |  |
| **Over all** | 19 | 1.06  [0.97, 1.15] | 0.20 | <0.01 | 68 | 20 | 1.06  [0.95, 1.18] | 0.29 | <0.01 | 61 | 19 | 1.04  [0.89, 1.20] | 0.65 | <0.01 | 64 | 19 | 1.07  [0.96, 1.20] | 0.24 | <0.01 | 58 | 19 | 1.03  [0.85, 1.24] | 0.77 | <0.01 | 64 |
| **Ethnic** |  |  |  |  |  |  |  |  |  |  |  |  |  |  |  |  |  |  |  |  |  |  |  |  |  |
| Asian | 11 | 1.07  [0.95, 1.20] | 0.30 | <0.01 | 77 | 11 | 1.04  [0.88, 1.23] | 0.62 | <0.01 | 66 | 11 | 1.12  [0.94, 1.33] | 0.20 | <0.01 | 70 | 11 | 1.00  [0.86, 1.16] | 0.98 | 0.02 | 52 | 11 | 1.12  [0.89, 1.42] | 0.33 | <0.01 | 72 |
| Caucasian | 8 | 1.04  [0.96, 1.12] | 0.32 | 0.09 | 44 | 9 | 1.09  [0.94, 1.25] | 0.27 | 0.02 | 57 | 8 | 0.82  [0.60, 1.13] | 0.22 | 0.07 | 46 | 8 | 1.16  [0.98, 1.38] | 0.08 | 0.01 | 62 | 8 | 0.86  [0.64, 1.17] | 0.34 | 0.11 | 40 |
| **Cancer type** |  |  |  |  |  |  |  |  |  |  |  |  |  |  |  |  |  |  |  |  |  |  |  |  |  |
| ESCC | 4 | 0.91  [0.76, 1.09] | 0.29 | 0.03 | 65 | 4 | 0.86  [0.74, 1.00] | 0.06 | 0.11 | 50 | 4 | 0.88  [0.64, 1.21] | 0.43 | 0.08 | 55 | 4 | 0.88  [0.73, 1.07] | 0.21 | 0.25 | 27 | 4 | 0.83  [0.55, 1.26] | 0.39 | 0.03 | 66 |
| HNSCC | 2 | 1.17  [0.88, 1.56] | 0.27 | 0.03 | 78 | 2 | 1.21  [0.88, 1.65] | 0.23 | <0.01 | 74 | 2 | 1.18  [0.69, 2.03] | 0.54 | 0.19 | 42 | 2 | 1.19  [0.90, 1.57] | 0.21 | 0.09 | 66 | 2 | 1.28  [0.67, 2.45] | 0.45 | 0.13 | 56 |
| NPC | 2 | **1.39**  **[1.12, 1.72]** | <0.01 | 0.88 | 0 | 2 | 1.18  [0.77, 1.81] | 0.45 | 0.59 | 0 | 2 | **1.77**  **[1.31, 2.40]** | <0.01 | 0.92 | 0 | 2 | 0.91  [0.58, 1.43] | 0.68 | 0.51 | 0 | 2 | **1.64**  **[1.02, 2.61]** | 0.04 | 0.57 | 0 |
| OSCC | 2 | 0.93  [0.78, 1.11] | 0.41 | 0.84 | 0 | 3 | 0.94  [0.76, 1.17] | 0.58 | 0.56 | 0 | 2 | 0.83  [0.64, 1.07] | 0.15 | 0.68 | 0 | 2 | 1.13  [0.82, 1.56] | 0.47 | 0.51 | 0 | 2 | 0.93  [0.63, 1.38] | 0.73 | 0.48 | 0 |
| PTC | 7 | 1.02  [0.95, 1.10] | 0.58 | 0.37 | 8 | 7 | 1.05  [0.90, 1.23] | 0.50 | 0.06 | 50 | 7 | 0.94  [0.76, 1.17] | 0.58 | 0.03 | 57 | 7 | 1.09  [0.89, 1.32] | 0.40 | <0.01 | 66 | 7 | 0.93  [0.75, 1.14] | 0.48 | 0.16 | 35 |
| **miR-149: rs2292832 (C/T)** | | | | |  |  |  |  |  |  |  |  |  |  |  |  |  |  |  |  |  |  |  |  |  |
| **Over all** | 4 | 0.97  [0.82, 1.15] | 0.72 | 0.02 | 68 | 5 | 0.98  [0.72, 1.32] | 0.88 | <0.01 | 75 | 4 | 0.91  [0.80, 1.04] | 0.17 | 0.45 | 0 | 4 | 0.99  [0.66, 1.48] | 0.97 | <0.01 | 78 | 4 | 0.96  [0.60, 1.52] | 0.86 | <0.01 | 79 |
| **Ethnic** |  |  |  |  |  |  |  |  |  |  |  |  |  |  |  |  |  |  |  |  |  |  |  |  |  |
| Asian | 3 | 0.99  [0.75, 1.31] | 0.95 | 0.01 | 77 | 3 | 1.05  [0.50, 2.20] | 0.91 | <0.01 | 86 | 3 | 0.92  [0.79, 1.06] | 0.24 | 0.27 | 24 | 3 | 1.04  [0.50, 2.19] | 0.91 | <0.01 | 83 | 3 | 1.03  [0.48, 2.19] | 0.94 | <0.01 | 85 |
| Caucasian | 1 | N/A | N/A | N/A | N/A | 2 | 0.99  [0.85, 1.14] | 0.88 | 0.87 | 0 | 1 | N/A | N/A | N/A | N/A | 1 | N/A | N/A | N/A | N/A | 1 | N/A | N/A | N/A | N/A |
| **Cancer type** |  |  |  |  |  |  |  |  |  |  |  |  |  |  |  |  |  |  |  |  |  |  |  |  |  |
| HNSCC | 2 | 1.13  [0.79, 1.61] | 0.50 | 0.04 | 77 | 2 | 1.52  [0.58, 3.98] | 0.39 | <0.01 | 88 | 2 | 1.00  [0.78, 1.27] | 0.98 | 0.23 | 31 | 2 | 1.52  [0.60, 3.85] | 0.38 | <0.01 | 86 | 2 | 1.47  [0.51, 4.25] | 0.47 | <0.01 | 87 |
| OSCC | 1 | N/A | N/A | N/A | N/A | 2 | 0.94  [0.72, 1.22] | 0.64 | 0.36 | 0 | 1 | N/A | N/A | N/A | N/A | 1 | N/A | N/A | N/A | N/A | 1 | N/A | N/A | N/A | N/A |
| **miR-196a2: rs11614913 (C/T)** | | | | | |  |  |  |  |  |  |  |  |  |  |  |  |  |  |  |  |  |  |  |  |
| **Over all** | 11 | 1.01  [0.92, 1.11] | 0.84 | <0.01 | 69 | 13 | 1.10  [0.99, 1.22] | 0.08 | 0.04 | 46 | 11 | 0.90  [0.74, 1.09] | 0.27 | <0.01 | 76 | 11 | **1.12**  **[1.03, 1.22]** | **0.01** | 0.07 | 42 | 11 | 1.00  [0.79, 1.25] | 0.97 | <0.01 | 76 |
| **Ethnic** |  |  |  |  |  |  |  |  |  |  |  |  |  |  |  |  |  |  |  |  |  |  |  |  |  |
| Asian | 10 | 1.01  [0.90, 1.12] | 0.91 | 0.0002 | 72 | 10 | 1.08  [0.99, 1.18] | 0.10 | 0.01 | 58 | 10 | 0.89  [0.72, 1.11] | 0.29 | <0.01 | 78 | 10 | **1.14**  **[1.01, 1.22]** | 0.03 | 0.04 | 48 | 10 | 0.99  [0.76, 1.29] | 0.94 | <0.01 | 78 |
| Caucasian | 1 | N/A | N/A | N/A | N/A | 2 | 1.09  [0.93, 1.28] | 0.26 | 0.69 | 0 | 1 | N/A | N/A | N/A | N/A | 1 | N/A | N/A | N/A | N/A | 1 | N/A | N/A | N/A | N/A |
| **Cancer type** |  |  |  |  |  |  |  |  |  |  |  |  |  |  |  |  |  |  |  |  |  |  |  |  |  |
| ESCC | 5 | 0.92  [0.76, 1.12] | 0.42 | 0.0008 | 79 | 5 | 1.00  [0.88, 1.14] | 0.97 | 0.11 | 48 | 5 | 0.75  [0.49, 1.13] | 0.17 | <0.001 | 84 | 5 | 1.09  [0.95, 1.25] | 0.25 | 0.65 | 0 | 5 | 0.79  [0.49, 1.27] | 0.34 | <0.01 | 83 |
| HNSCC | 1 | N/A | N/A | N/A | N/A | 2 | 1.13  [0.98, 1.31] | 0.09 | 0.72 | 0 | 1 | N/A | N/A | N/A | N/A | 1 | N/A | N/A | N/A | N/A | 1 | N/A | N/A | N/A | N/A |
| OSCC | 3 | **1.16**  **[1.02, 1.32]** | 0.02 | 0.92 | 0 | 4 | **1.32**  **[1.11, 1.56]** | <0.01 | 0.12 | 48 | 3 | 0.99  [0.79, 1.23] | 0.90 | 0.54 | 0 | 3 | **1.54**  **[1.10, 2.18]** | 0.01 | 0.11 | 55 | 3 | **1.43**  **[1.09, 1.89]** | 0.01 | 0.83 | 0 |
| **miR-423: rs6505162 (C/A)** | | | | | |  |  |  |  |  |  |  |  |  |  |  |  |  |  |  |  |  |  |  |  |
| **Over all** | 4 | 1.12  [1.00, 1.26] | 0.05 | 0.25 | 26 | 4 | 1.13  [0.98, 1.31] | 0.09 | 0.46 | 0 | 4 | 1.23  [0.95, 1.61] | 0.12 | 0.17 | 41 | 4 | 1.10  [0.94, 1.27] | 0.23 | 0.66 | 0 | 4 | 1.28  [0.96, 1.71] | 0.09 | 0.13 | 47 |
| **Ethtic** |  |  |  |  |  |  |  |  |  |  |  |  |  |  |  |  |  |  |  |  |  |  |  |  |  |
| Asian | 2 | 1.10  [0.94,1.27] | 0.23 | 0.35 | 0 | 2 | 1.09  [0.90, 1.32] | 0.37 | 0.54 | 0 | 2 | 1.20  [0.87, 1.64] | 0.27 | 0.17 | 47 | 2 | 1.05  [0.86, 1.29] | 0.62 | 0.68 | 0 | 2 | 1.24  [0.87, 1.76] | 0.24 | 0.16 | 49 |
| **miR-499a: rs3746444 (T/C)** | | | | | | |  |  |  |  |  |  |  |  |  |  |  |  |  |  |  |  |  |  |  |
| **Over all** | 8 | 0.99  [0.83, 1.20] | 0.45 | <0.01 | 81 | 9 | 0.94  [0.78, 1.13] | 0.08 | <0.01 | 78 | 8 | 1.12  [0.90, 1.41] | 0.31 | 0.06 | 48 | 8 | 0.95  [0.78, 1.15] | 0.10 | <0.01 | 75 | 8 | 1.06  [0.71, 1.58] | 0.46 | 0.03 | 56 |
| **Ethnic** |  |  |  |  |  |  |  |  |  |  |  |  |  |  |  |  |  |  |  |  |  |  |  |  |  |
| Asian | 7 | 1.02  [0.81, 1.27] | 0.88 | <0.01 | 83 | 7 | 0.99  [0.78, 1.27] | 0.96 | <0.01 | 81 | 7 | 1.17  [0.89, 1.55] | 0.27 | 0.04 | 55 | 7 | 0.98  [0.78, 1.22] | 0.83 | <0.01 | 76 | 7 | 1.11  [0.65, 1.88] | 0.71 | 0.02 | 61 |
| Caucasian | 1 | N/A | N/A | N/A | N/A | 2 | **0.80**  **[0.69, 0.94]** | 0.005 | 0.54 | 0 | 1 | N/A | N/A | N/A | N/A | 1 | N/A | N/A | N/A | N/A | 1 | N/A | N/A | N/A | N/A |
| **Cancer type** |  |  |  |  |  |  |  |  |  |  |  |  |  |  |  |  |  |  |  |  |  |  |  |  |  |
| ESCC | 2 | **0.80**  **[0.66, 0.98]** | 0.03 | 0.75 | 0 | 2 | 0.79  [0.62, 1.00] | 0.05 | 0.83 | 0 | 2 | 0.64  [0.36, 1.14] | 0.13 | 0.93 | 0 | 2 | 0.81  [0.63, 1.04] | 0.10 | 0.80 | 0 | 2 | 0.60  [0.33, 1.08] | 0.09 | 0.88 | 0 |
| OSCC | 3 | 1.21  [0.64, 2.28] | 0.56 | <0.01 | 52 | 4 | 0.97  [0.83, 1.13] | 0.68 | <0.01 | 90 | 3 | 2.29  [0.71, 7.37] | 0.17 | 0.10 | 57 | 3 | 1.09  [0.55, 2.16] | 0.80 | <0.01 | 91 | 3 | 2.40  [0.64, 8.98] | 0.19 | 0.06 | 65 |
| **miR-608: rs4919510 (G/C)** | | | | | |  |  |  |  |  |  |  |  |  |  |  |  |  |  |  |  |  |  |  |  |
| **Over all** | 4 | 0.91  [0.80, 1.04] | 0.17 | <0.01 | 75 | 4 | 0.93  [0.79, 1.11] | 0.44 | 0.04 | 64 | 4 | **0.82**  **[0.68, 0.98]** | 0.08 | 0.07 | 58 | 4 | 0.99  [0.89, 1.11] | 0.88 | 0.21 | 33 | 4 | 0.81  [0.63, 1.06] | 0.12 | 0.01 | 72 |
| **Cancer type** |  |  |  |  |  |  |  |  |  |  |  |  |  |  |  |  |  |  |  |  |  |  |  |  |  |
| NPC | 2 | **0.83**  **[0.75, 0.91]** | <0.01 | 0.8 | 0 | 2 | **0.81**  **[0.70, 0.94]** | <0.01 | 0.73 | 0 | 2 | **0.74**  **[0.63, 0.86]** | <0.01 | 0.92 | 0 | 2 | 0.88  [0.75, 1.04] | 0.13 | 0.73 | 0 | 2 | **0.68**  **[0.57, 0.82]** | <0.01 | 0.77 | 0 |

SNPs: single nucleotide polymorphisms; n: number of cohorts; OR: odd ratio; CI: confidence interval; P-H: p value of heterogeneity; ESCC: esophageal squamous cell carcinoma ;HNSCC: head and neck squamous cell carcinoma; NPC: nasopharyngeal carcinoma ; OSCC: oral squamous cell carcinoma; PTC: papillary thyroid carcinoma.
